# The Topographical Mapping in Drosophila Central Complex Network and Its Signal Routing

**DOI:** 10.3389/fninf.2017.00026

**Published:** 2017-04-10

**Authors:** Po-Yen Chang, Ta-Shun Su, Chi-Tin Shih, Chung-Chuan Lo

**Affiliations:** ^1^Institute of Systems Neuroscience, National Tsing Hua UniversityHsinchu, Taiwan; ^2^Department of Applied Physics, Tunghai UniversityTaichung, Taiwan; ^3^National Center for High-Performance ComputingHsunchu, Taiwan; ^4^Department of Life Science, National Tsing Hua UniversityHsinchu, Taiwan

**Keywords:** central complex, neural networks, *Drosophila*, protocerebral bridge, topographical mapping

## Abstract

Neural networks regulate brain functions by routing signals. Therefore, investigating the detailed organization of a neural circuit at the cellular levels is a crucial step toward understanding the neural mechanisms of brain functions. To study how a complicated neural circuit is organized, we analyzed recently published data on the neural circuit of the *Drosophila* central complex, a brain structure associated with a variety of functions including sensory integration and coordination of locomotion. We discovered that, except for a small number of “atypical” neuron types, the network structure formed by the identified 194 neuron types can be described by only a few simple mathematical rules. Specifically, the topological mapping formed by these neurons can be reconstructed by applying a generation matrix on a small set of initial neurons. By analyzing how information flows propagate with or without the atypical neurons, we found that while the general pattern of signal propagation in the central complex follows the simple topological mapping formed by the “typical” neurons, some atypical neurons can substantially re-route the signal pathways, implying specific roles of these neurons in sensory signal integration. The present study provides insights into the organization principle and signal integration in the central complex.

## Introduction

Brain functions originate from interactions between neurons, which are strongly regulated by signal routing in neural networks. To fully understand the neural mechanisms that underlie brain functions, one must investigates the detailed organization of a neural circuit in single-neuron resolution. Recent studies on *Drosophila* connectome mapping (Chiang et al., 2011; Lin H.-H. et al., 2013; Takemura et al., 2013; Shih et al., 2015) provided an opportunity for high-resolution neural circuit analysis. Among these studies, the release of the neuron innervation map in the central complex (Lin C.-Y. et al., 2013; Wolff et al., 2015) is of particular interest for its potential role in sensory-motor integration and memory.

The central complex is a brain structure that commonly exists in arthropods (Power, 1943; Loesel et al., 2002; Homberg, 2008). In *Drosophila*, the central complex consists of five neuropils: protocerebral bridge (PB), fan-shaped body (FB), ellipsoid body (EB), and a pair of noduli (NO) (Supplementary Figure S1). The central complex is anatomically separated from other brain regions by a glia sheath and makes relatively fewer direct contacts with sensory or motor centers. Instead, the central complex communicates with other brain regions through the neighboring neuropils including lateral triangle (LTR), caudalcentral protocerebrum (CCP), caudal ventrolateral protocerebrum (CVLP), ventromedial protocerebrum (VMP), and inferior dorsofrontal protocerebrum (IDFP) (also called ventral body or lateral accessory lobe) (Hanesch et al., 1989; Renn et al., 1999; Young and Armstrong, 2010b).

A number of studies have revealed the diversity in the functions of the central complex (Wessnitzer and Webb, 2006; Lin C.-Y. et al., 2013; Strausfeld and Hirth, 2013; Seelig and Jayaraman, 2015). Specifically, the functions of the central complex can be divided into three categories:

1. Sensory information integration. Studies on locusts revealed that some PB neurons are selective to polarized light in the sky in order to guide spatial orientation (Heinze and Homberg, 2007, 2009; Heinze et al., 2009; Heinze and Reppert, 2011; Homberg et al., 2011). A recent study demonstrated the role of EB in landmark orientation and angular path integration (Seelig and Jayaraman, 2015).
2. Coordination of locomotion and high-level behavior. Experiments have shown that lesions in the central complex lead to various abnormalities in locomotion, such as walking, flying, frequency of movements, and turning control (Strauss and Heisenberg, 1993; Martin et al., 1999; Strauss, 2002; Ridgel et al., 2006; Triphan et al., 2010). Other studies also demonstrated the roles of the central complex in high level behavior such as pursuing, responses to ethanol, courtship behavior, as well as sleep and arousal (Strauss and Pichler, 1998; Sakai and Kitamoto, 2006; Kong et al., 2010; Ueno et al., 2012).
3. Memory. Although memory in *Drosophila* has long been associated with the mushroom body, recent studies have begun to link the central complex with some types of memory (Wu et al., 2007). Short-term memory of visual patterns has been localized to specific types of FB neurons (Liu et al., 2006; Neuser et al., 2008).

What are the neural circuit mechanisms that underlie such diverse functionality of the central complex? While the answer remains elusive, accumulating anatomical evidence of the central complex indicates the highly structured nature of the neural circuit organization (Hanesch et al., 1989; Heinze and Homberg, 2008; Young and Armstrong, 2010a,b), which provides insights into the wiring principle of the central complex.

**Figure 1:**
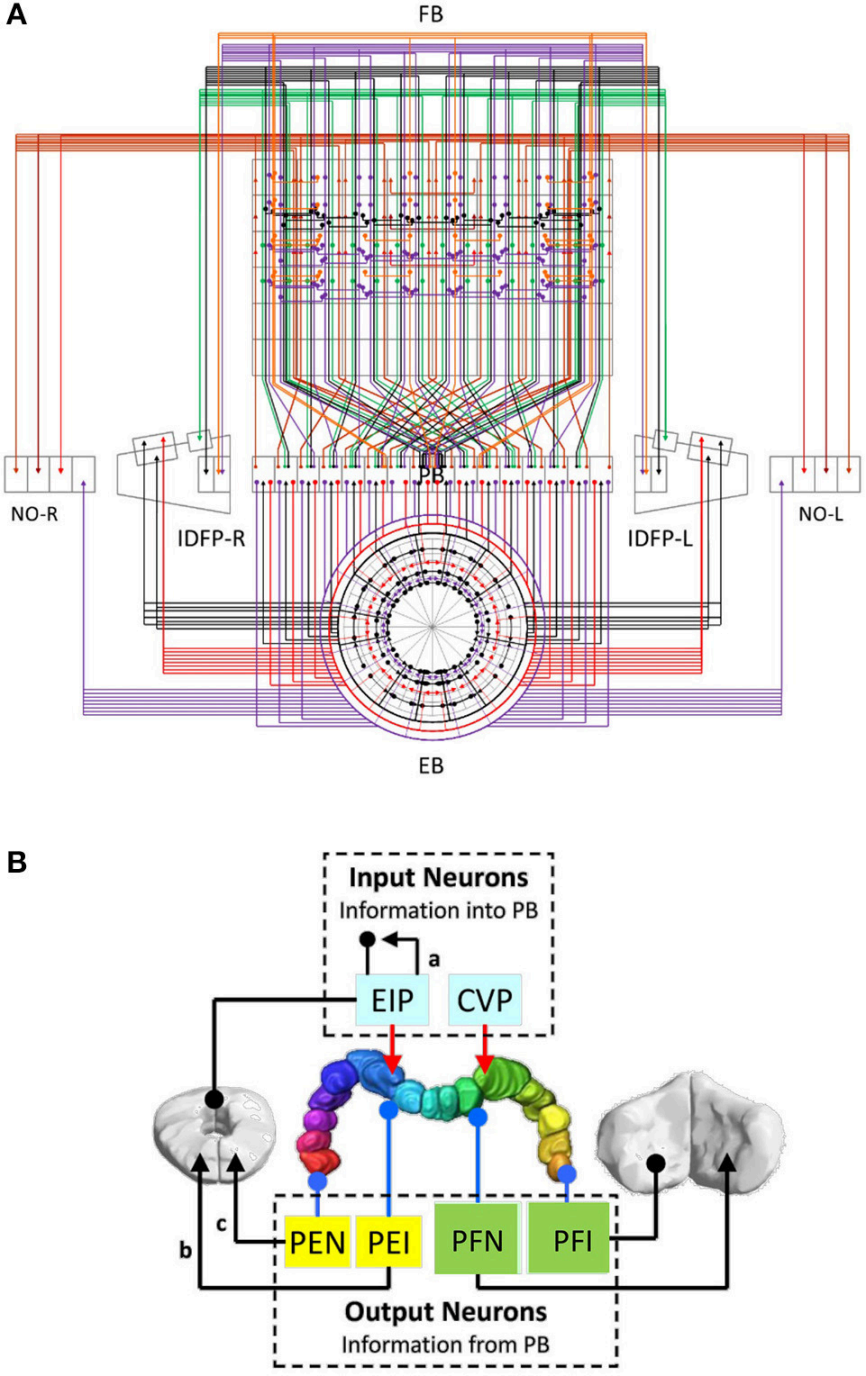
**The partial central complex network formed by PB-innervating neurons. (A)** The innervation diagram of the network. Each neuron type is represented by a color line with arrowheads for axonal innervation and solid circles for dendritic innervation. Each color indicates a specific neuron class. **(B)** The large-scale circuit diagram of the central complex showing the relationship between neuron classes. The arrowheads and solid circles are defined as in **(A)**. EIP and CVP are input neuron classes for their axonal innervation in PB while other classes are classified as output neurons for their dendritic innervation in PB. The PB local neuron class is not shown. The present study focuses on the recurrent circuits formed by EIP, PEI, and PEN.

In the present study, we focused on a PB related circuit because among the five neuropils of the central complex, PB has the most detailed wiring diagram available, as described in two recent optical imaging studies (Lin C.-Y. et al., 2013; Wolff et al., 2015). Due to the limitation of the imaging resolution, these studies described the connectivity between single neurons and glomeruli rather than the synaptic connections between neurons. However, the studies still revealed intriguing connectivity patterns, which suggest an “electrical-circuit-like” wiring diagram in the central complex (Lin C.-Y. et al., 2013; Wolff et al., 2015). Such finding implies that the operational principles of the central complex may be inferred by mathematical analysis of its circuit organization. The PB related circuit consists of several classes of neurons and each class is characterized by unique innervation patterns between neuropils. The circuit has two general figures: (1) two feedback loops. There are several classes of neurons sending information back and forth between PB and EB as well as between PB and IDFP. (2) Topographical mapping. In general, two neurons that have their dendritic projections in the adjacent regions also project their axons to the neighbor regions in the target neuropils (Supplementary Presentation S1).

We set out to derive a mathematical representation of the PB circuit described in Lin C.-Y. et al. (2013). We found that the highly structured and topological innervation patterns can be produced by simple mathematical rules. However, a small portion of the neurons does not follow the regularity and cannot be predicted by the rules. We further investigated these “atypical” neurons and found that two of them substantially reroute signal propagation in the network. The significance of rerouting and its potential functions are also discussed in this paper.

## Methods

### Neuropils and subunits

In the present study we analyzed the central complex circuit based on the data published in Lin C.-Y. et al. (2013). The authors used MARCM (mosaic analysis with a repressible cell marker) technique to collect images of neurons that innervate PB in *Drosophila*. They further determined the accurate divisions (called subunits) of each neuropil in the central complex and then recorded the innervation sites for the axons and dendrites of each neuron. A total of 662 images were collected. The neuron IDs were listed in Lin C.-Y. et al. (2013) and most the images are available in the FlyCircuit database (http://www.flycircuit.tw/).

Many neuropils in *Drosophila* consist of multiple glomeruli, which are the destinations of neuron processes and are characterized by aggregated synapses. Based on the distributions of glomeruli, Lin et al. further divided each of the five neuropils of the central complex and the four pairs of associated neuropils into 154 subunits (Lin C.-Y. et al., 2013) (Supplementary Figures S1, S2). In the present study we focused on the PB-innervating neurons described in Lin C.-Y. et al. (2013) as they innervate most of the subunits in the central complex. The associated neuropils IDFP, CCP, CVLP, and VMP, which are divided into 18 subunits in total, are not part of the central complex but receive innervations from many PB-innervating neurons. Therefore, these neuropils are included in the analysis (Supplementary Figure S1).

### Neuron classification and naming

In Lin C.-Y. et al. (2013), the authors classified all the 662 neurons into 194 types, and each type of neurons have unique innervation patterns at the subunit levels. The original classification in Lin C.-Y. et al. (2013) was hierarchically organized with superclass on top, followed by class, family, and then type at the lowest rank. For the sake of simplicity, here we used only two levels of classification: class (corresponds to superclass in Lin C.-Y. et al., 2013) and type. The neuron class is defined based on the innervation patterns at the neuropil level while the neuron type is defined based on the innervation at the subunit level. For example, if neurons A and B both project from neuropils 1 to 2, but innervate different subunits, these two neurons belong to the same class but they are of two different types. If the neuron C projects from neuropils 2 to 3, it belongs to another class which is different from that of the neurons A and B. In general, neurons in the same class are characterized by similar morphology. Note that due to the large size of FB, it is divided into six horizontal layers and each layer contains eight subunits (Lin C.-Y. et al., 2013). Neurons innervating different layers of FB are also classified into different classes. Check Supplementary Presentation S1 for the detailed innervation patterns of each neuron class and its types. Each neuron is named by its class and type (Supplementary Table S1). For example, EIP10 represents the neuron of type number 10 in the EIP class. Except for the PB LN class, which represents the PB local neurons, the class names are given based on the neuropils innervated by the neurons with an order indicating the flow of the information. For example, the EIP class represents neurons that innervate EB with dendritic domains and innervate IDFP and PB with axonal domains. See Supplementary Table S1 for a detailed comparison between the naming systems used in the present study and in Lin C.-Y. et al. (2013).

### Neuron innervation vectors and generation matrices

We represent the innervation pattern of each type of neurons by a 154-dimensional vector. Each dimension (element) of the vector corresponds to one of the subunits in the central complex and associated neuropils. See Supplementary Figure S2 for the list and order of the subunits, and Supplementary Tables S2–S3 for the complete innervation tables of each neuron. In each dimension, the innervation type of the neurons is indicated by a number between 0 and 3, with 0 for no innervation, 1 for dendrites, 2 for axons, and 3 for axon/dendrite coexistence. For example, CVP1 neuron innervates subunits R1 and R2 in PB, L in CCP, and d-L in VMP, with axon in PB and dendrites in CCP and VMP. The innervation vector of the CVP1 neuron can be expressed by

(1) $$\begin{array}{llll} & PBR8-R1 & CCP & VMP\\ CVP1= & (\overset{}{\overset{︷}{0\;0\;0\;0\;0\;0\;2\;2}}\ldots \ldots \ldots & \overset{}{\overset{︷}{0\;1}}\ldots . & \overset{}{\overset{︷}{0\;0\;1\;0}}). \end{array}$$

Besides the elements explicitly indicated above, all other elements (represented by the dots) in the vector are zero. We found that neurons under the same class are often represented by very similar innervation vectors with shifted elements in some neuropils. For example, the innervation vector of CVP2 neuron is

(2) $$\begin{array}{llll} & \;PBR8-R1 & CCP & VMP\\ CVP2= & (\overset{}{\overset{︷}{0\;0\;0\;0\;0\;2\;2\;0}}\ldots \ldots \ldots & \overset{}{\overset{︷}{0\;1}}\;\ldots . & \overset{}{\overset{︷}{0\;0\;1\;0}}). \end{array}$$

where the innervations remain the same as CVP1 neuron in CCP and VMP, but shifted to the left by one element in PB. A similar trend can be observed for CVP3–CVP7 neurons, which all have a left-shifted innervation pattern in PB with respect to the lower-numbered neuron types and identical innervation patterns in CCP and VMP. Based on the observations, we can apply a generation matrix, or generator, to an initial neuron (CVP1 in the case mentioned above) to generate the innervation vectors for other neuron types in the same class. The generator can be constructed using a permutation matrix in which only a single “1” is presented in each column and row, and the rest of the elements are 0's. To construct generators for different neuron classes, we need three major types of permutation matrices:

1. Translation matrix (**T**): It shifts every number in a vector equally by one or several elements. For example, the matrix *T*^(1, 4)^ below shifts every number in the vector *a* up by one element (equivalent to shifting to the left if the vector is represented as a row vector):

(3) $$T^{\left(1,4\right)}\times a=\left[\begin{array}{cccc} 0 & 1 & 0 & 0\\ 0 & 0 & 1 & 0\\ 0 & 0 & 0 & 1\\ 1 & 0 & 0 & 0 \end{array}\right]\left[\begin{array}{c} 0\\ 1\\ 2\\ 3 \end{array}\right]=\left[\begin{array}{c} 1\\ 2\\ 3\\ 0 \end{array}\right]$$

where the superscript (1,4) indicates a one-element shift for a four-dimensional vector.

2. Mirror matrix (**M**): It reverses the order of the elements in a vector, as if we take a mirror image of the innervation pattern with respect to the midline. The matrix **M** below demonstrates an example mirror matrix.

(4) $$\text{M}\times a=\left[\begin{array}{cccc} 0 & 0 & 0 & 1\\ 0 & 0 & 1 & 0\\ 0 & 1 & 0 & 0\\ 1 & 0 & 0 & 0 \end{array}\right]\left[\begin{array}{c} 0\\ 1\\ 2\\ 3 \end{array}\right]=\left[\begin{array}{c} 3\\ 2\\ 1\\ 0 \end{array}\right]$$

However, due to the arrangement of the subunit in IDFP, NO, and VMP, the mirror matrix of these neuropils requires special forms. Taking IDFP, for example, its subunits are arranged in the following order:

$$\begin{array}{l} \text{IDFP vector = }(\text{HBm}-\text{L},\text{ HBI-L},\text{ DSB-L},\text{ VSB-L},\\ \;↓\text{ midline}\\ \text{ RB-L},\text{ HBm-R},\text{ HBI-R},\text{ DSB-R},\\ \text{ VSB-R},\text{RB-R}) \end{array}$$

A mirror matrix should map innervations between HBm-R and HBm-L, HBI-R and HBI-L, and so on. Therefore, the mirror generator for IDFP takes the following form:

(5) $$\begin{array}{l} M_{IDFP}=M^{\left(2\right)}⊗I^{\left(5\right)}=\left[\begin{array}{cc} 0 & 1\\ 1 & 0 \end{array}\right]⊗\left[\begin{array}{ccccc} 1 & \; & \; & \; & \;\\ \; & 1 & \; & \; & \;\\ \; & \; & 1 & \; & \;\\ \; & \; & \; & 1 & \;\\ \; & \; & \; & \; & 1 \end{array}\right]\\ =\left[\begin{array}{cccccccccc} \; & \; & \; & \; & \; & 1 & \; & \; & \; & \;\\ \; & \; & \; & \; & \; & \; & 1 & \; & \; & \;\\ \; & \; & \; & \; & \; & \; & \; & 1 & \; & \;\\ \; & \; & \; & \; & \; & \; & \; & \; & 1 & \;\\ \; & \; & \; & \; & \; & \; & \; & \; & \; & 1\\ 1 & \; & \; & \; & \; & \; & \; & \; & \; & \;\\ \; & 1 & \; & \; & \; & \; & \; & \; & \; & \;\\ \; & \; & 1 & \; & \; & \; & \; & \; & \; & \;\\ \; & \; & \; & 1 & \; & \; & \; & \; & \; & \;\\ \; & \; & \; & \; & 1 & \; & \; & \; & \; & \; \end{array}\right], \end{array}$$

where ⊗ denotes the Kronecker product. The mirror matrix for NO and VMP also takes the similar form.

Identity matrix (**I**): In some neuropils, different neuron types in the same class innervate the same subunits without translation. This pattern (called “standing”) can be described by an identity matrix:

(6) $$I\times a=\left[\begin{array}{cccc} 1 & 0 & 0 & 0\\ 0 & 1 & 0 & 0\\ 0 & 0 & 1 & 0\\ 0 & 0 & 0 & 1 \end{array}\right]\times \left[\begin{array}{c} 0\\ 1\\ 2\\ 3 \end{array}\right]=\left[\begin{array}{c} 0\\ 1\\ 2\\ 3 \end{array}\right]$$

We describe how generators can be constructed by combining the three matrix types in the Results Section.

### Construction of the connection matrix

To analyze how the PB circuit supports signal propagation in the central complex, we first need to construct the connection matrix, or adjacency matrix for the 194 neuron types (See Supplementary Tables S4–S5 for the complete matrices). The matrix was constructed based on the hypothesis that a neuron innervating a given subunit with an axonal arbor forms synapses with a neuron which innervates the same subunit with a dendritic arbor. The hypothesis is based on the following considerations:

1. We estimated the envelopes that enclose the dendritic or axonal arbors in several subunits for a number of representative neurons and found the average size of the envelope to be 15.63 μm, which is comparable to the average size of the subunits (= 15.89 μm) in PB, FB, and EB (Supplementary Table S6). The result indicated that the neuronal arbors inside a subunit are spatially overlapped, suggesting a strong probability of synapse formation between dendritic and axonal processes that innervate the same subunit. We note that, however, a recent study on the rodent neocortex suggested that the proximity between axon and dendrite does not necessarily indicate synapse formation between them (Kasthuri et al., 2015). Considering the significant differences in the neuron morphology and circuit organization between insects and mammals, it remains to be tested whether this is the same case in *Drosophila* brain.
2. The subunits are basically glomeruli, which are known to be the places with a high density distribution of synapses. Moreover, the subunits were defined based on the Dlg (discs-large) immunology. Dlg proteins play an essential role in synaptic clustering of K^+^ channels and cell adhesion molecules (Tejedor et al., 1997).
3. In insect brains, neurons (often unipolar neurons) have their trunks (primary neurite) branch out into two or more neurites. Each neurite projects to a specific neuropil and often terminates in a glomerulus with arbors rich of spines/twigs or boutons, which are known to be the main locations of postsynaptic or presynaptic sites in the Drosophila brain, respectively (Schneider-Mizell et al., 2016). Moreover, many neurons only project to two glomeruli, one for dendrite and the other for axon. If these neurons do not form synapses in both glomeruli, no information can be received or sent by the neurons. Therefore, axonal projections forming synapses with dendrites in the same glomerulus has become a basic assumption and has also been demonstrated in various Drosophila studies (Träger et al., 2008; Olsen et al., 2010; Held et al., 2016). It has also been shown in the central complex (Lin C.-Y. et al., 2013) as well as in several other neuropils (Chou et al., 2010; Liang et al., 2013) that neuronal terminals in the glomeruli are expressed with large numbers of presynaptic and/or postsynaptic markers.

Our goal here is to analyze how different neuropils communicate with each other in the central complex. Hence, when we constructed the connection matrix, we excluded the PB local neurons, which only propagate signals within PB. Furthermore, most of the PB local neurons innervate nearly every PB subunit, leading to a nearly identical and uniform input to every subunit from all other subunits. Although the exact function of these local neurons is unknown, local neurons in many other nervous systems exhibit similar large-field coverages and are known to provide modulatory functions such as lateral inhibition, gain control, or normalization (Cook and McReynolds, 1998; Olsen and Wilson, 2008; Olsen et al., 2010). Hence, we hypothesized that due to the possible role in global modulation, these local neurons do not substantially bias the signal flows between individual subunits in different neuropils, and therefore can be excluded from the connection matrix.

### Network analysis

We performed several basic and novel analyses of the central complex networks based on the connection matrices described above. In the basic analyses we estimated the fundamental properties of the PB networks, including the characteristic path length, clustering coefficient, modularity, global efficiency, and small-worldness, by using Brain Connectivity Toolbox (BCT) (Rubinov and Sporns, 2010).

In the novel analyses we investigated the connection matrices at the different propagation levels (Lin et al., 2014). The analysis has been shown to reveal different efficiency in signal propagation between various networks of similar small-world properties. It also identified the functional modules associated with reflexive behaviors and complex/social behaviors of *C. elegans* (Lin et al., 2014). The propagation level is defined as the number of intermediate neurons making up a path between the given input (source) and output (destination) nodes. For example, a path I → A → B → O, which connects the neuron I and the neuron O via the intermediate neurons A and B, is a path of propagation level 2. Note that recurrent paths, which pass through the same neurons multiple times, also count. Therefore, the path I → A → B → A → B → O is counted as a level 4 path. We construct the connection matrix **M**_**l**_ = *m*(*i, j*)_*l*_ where *m*(*i, j*)_*l*_ indicates the number of paths that connect neurons *i* and *j* at the level *l*.

The advantage of analyzing the connection matrix **M**_*l*_ at high levels is that it reveals neuron pairs that are connected by strongly recurrent pathways, because *m*(*i, j*)_*l*_ increases rapidly when neurons *i* and *j* are connected through strongly recurrent circuits (Lin et al., 2014). Theoretical studies have suggested that recurrent neural networks are associated with a variety of complex functions, including working memory, perceptual decision, oscillation, and etc (Wang, 1999, 2002; Brunel, 2000; Laje and Buonomano, 2013). Therefore, examining information flows that go through strongly or weakly recurrent pathways in a network may provide insights into its functional significance from the theoretical perspective.

## Results

### Mathematical description of the innervation patterns of the central complex neurons

We first plotted the circuit diagram of the central complex for the PB-innervating neurons described in Lin C.-Y. et al. (2013). The circuit diagram showed how neurons innervate the 154 subunits (Figure 1A and Supplementary Figure S1) in 12 neuropils (4 from the central complex and 8 from associated regions) (Lin C.-Y. et al., 2013). We found that the neurons exhibit highly structural and regular innervation patterns in the central complex (Figure 1A). We adopted a simplified version of the neuron classification proposed in Lin C.-Y. et al. (2013) by classifying all neurons into 13 classes based on their innervation patterns (Supplementary Table S1). Among the 13 classes, EIP, CIVP, and CVP can be considered as the input neuron classes of PB, while PEI, PEN, and PFNs (3 classes) and PFIs (4 classes) are output classes, and PBLN is the class of local neurons of PB (Figure 1B). Furthermore, the PEN and PEI neurons have axonal projections in EB, where EIP neurons' dendritic domains are located. These neurons are likely to form feedback loops, which suggest a strong interaction between PB and EB (Figure 1B). The 13 classes can be further divided into 194 neuron types according to their innervation patterns in the 154 subunits of the 13 neuropils (see Methods for the naming of the neuron classes and types). Each neuron type can be represented by a 154-dimensioned vector, which uniquely indicates the neuron's innervation pattern (see Methods).

**Figure 2:**
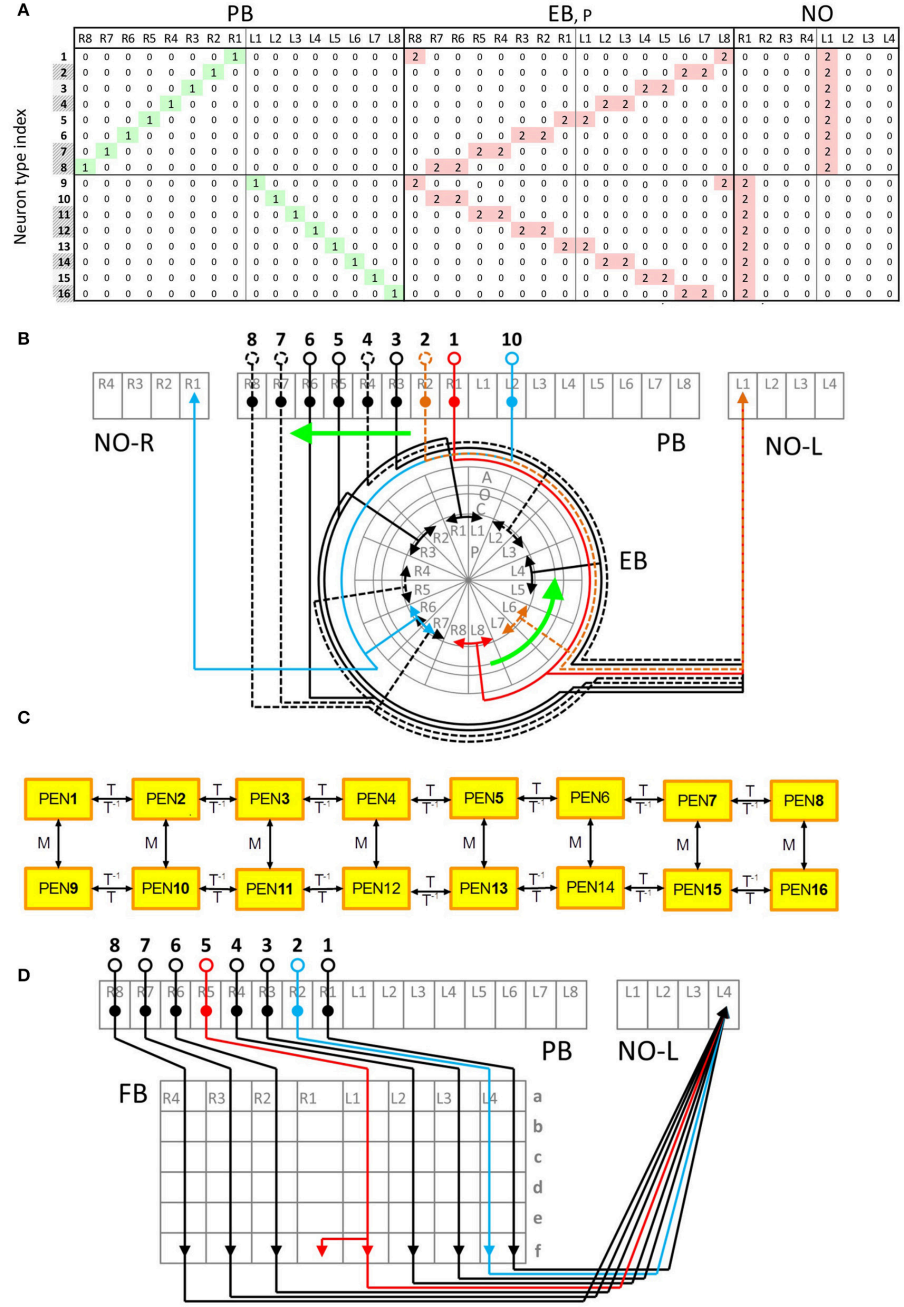
**Typical and atypical innervation patterns of neurons in the central complex. (A)** The innervation table for the PEN class. Each row represents the innervation vector of a given PEN neuron type while each column indicates how a subunit is innervated by different neuron types (0 for no innervation, 1 for dendrite and 2 for axon). Shaded neuron type indices indicate the types that were not observed but predicted in Lin C.-Y. et al. (2013). This neuron class demonstrates a typical (or regular) innervation pattern. **(B)** The innervation diagram of the PEN class. Neuron types are labeled by black numbers and the subunits are in gray. The relationship between the neuron types PEN1-PEN8 (numbers 1-8) can be described as shifting. The same relationship is observed for PEN9-PEN16. In addition, PEN9-PEN16 are a mirroring of PEN1-PEN8, respectively. The arrowheads represent axons while the solid circles indicate dendrites. The somas are represented by the empty circles. Dashed lines are neurons predicted in Lin C.-Y. et al. (2013). **(C)** A generator diagram of the PEN class showing how generators can be used to produce one neuron type from others. The PEN class demonstrates a regular (or typical) innervation pattern with which all neuron types can be generated from one initial neuron by recursive application of T or M generators. The rightward arrows indicate the effect of the generators labeled above the arrows while the leftward arrows is for the generators labeled below. **(D)** Atypical innervation patterns of the PFN-FfN4 class. The type 2 neuron does not make a shift with respect to the type 1 as expected, but innervates the same FB unit (FBf-L4) as type 1. The type 5 neuron innervates two adjacent FB subunits while other neurons only innervate one.

Within a class, neurons exhibit very similar innervation patterns. Taking the PEN neurons for example, each neuron extends its dendrite to a single PB subunit and projects axons to two neighboring EB subunits and one NO subunit (Figures 2A,B). The subunits innervated by the neuron type PEN2 are adjacent to those innervated by PEN1. The regularity allows us to construct generators (generation matrices) which describe the relationship between neuron types in the central complex. The innervation vector of each neuron type can be generated by applying the generators on the innervation vectors of other neuron types. We constructed the generators based on the following rules observed in most central complex neurons:

1. **Isomorphism:** The neurons of the same class innervate the same subunits with the same polarity in a neuropil. There are a few exceptions, which are described later.
2. **Mirroring:** For any given neuron type in the central complex, there exists a contralateral neuron type which is the reflection of the given neuron type with respect to the midline.
3. **Translation:** Within the same class, the innervation pattern of one neuron type can be produced from that of another neuron type by shifting the innervated subunits to the adjacent ones. In general, the neighboring neurons in the same class can be obtained by shifting one subunit in PB and FB while shifting two subunits in EB, and these rules apply to all classes. There are a few exceptions, which are described later.

Due to the preservation of the translation rule across classes, we can construct one local generator that works for every class in a given neuropil. For example, for the neuropil PB, the translation generator (shift matrix) **T**_PB_ shifts every PB-innervating neuron by one subunit:

(7) $$\begin{array}{l} T_{PB}\times PB_{R1}=T^{\left(1,16\right)}\times {\text{PB}}_{R1}=\;\left[\begin{array}{cccccccccccccccc} \; & 1 & \; & \; & \; & \; & \; & \; & \; & \; & \; & \; & \; & \; & \; & \;\\ \; & \; & 1 & \; & \; & \; & \; & \; & \; & \; & \; & \; & \; & \; & \; & \;\\ \; & \; & \; & 1 & \; & \; & \; & \; & \; & \; & \; & \; & \; & \; & \; & \;\\ \; & \; & \; & \; & 1 & \; & \; & \; & \; & \; & \; & \; & \; & \; & \; & \;\\ \; & \; & \; & \; & \; & 1 & \; & \; & \; & \; & \; & \; & \; & \; & \; & \;\\ \; & \; & \; & \; & \; & \; & 1 & \; & \; & \; & \; & \; & \; & \; & \; & \;\\ \; & \; & \; & \; & \; & \; & \; & 1 & \; & \; & \; & \; & \; & \; & \; & \;\\ \; & \; & \; & \; & \; & \; & \; & \; & 1 & \; & \; & \; & \; & \; & \; & \;\\ \; & \; & \; & \; & \; & \; & \; & \; & \; & 1 & \; & \; & \; & \; & \; & \;\\ \; & \; & \; & \; & \; & \; & \; & \; & \; & \; & 1 & \; & \; & \; & \; & \;\\ \; & \; & \; & \; & \; & \; & \; & \; & \; & \; & \; & 1 & \; & \; & \; & \;\\ \; & \; & \; & \; & \; & \; & \; & \; & \; & \; & \; & \; & 1 & \; & \; & \;\\ \; & \; & \; & \; & \; & \; & \; & \; & \; & \; & \; & \; & \; & 1 & \; & \;\\ \; & \; & \; & \; & \; & \; & \; & \; & \; & \; & \; & \; & \; & \; & 1 & \;\\ \; & \; & \; & \; & \; & \; & \; & \; & \; & \; & \; & \; & \; & \; & \; & 1\\ 1 & \; & \; & \; & \; & \; & \; & \; & \; & \; & \; & \; & \; & \; & \; & \; \end{array}\right]\;\left[\begin{array}{c} 0\\ 0\\ 0\\ 0\\ 0\\ 0\\ 0\\ 1\\ 0\\ 0\\ 0\\ 0\\ 0\\ 0\\ 0\\ 0 \end{array}\right]=\left[\begin{array}{c} 0\\ 0\\ 0\\ 0\\ 0\\ 0\\ 1\\ 0\\ 0\\ 0\\ 0\\ 0\\ 0\\ 0\\ 0\\ 0 \end{array}\right]\\ \;=\;{\text{PB}}_{R2} \end{array}$$

where PB_*n*_ indicates the PB portion of an innervation vector of a neuron which projects to the ‘subunit *n* in PB. The same convention is used for the portion of innervation vectors in other neuropils. By constructing and combining the translation generators for every neuropil, we can obtain a global translation generator *T* for the central complex:

(8) $$T=T_{PB}⊕T_{FB}⊕T_{EB}⊕I_{NO}⊕I_{IDFP}⊕I_{CCP}⊕I_{CVLP}⊕I_{\text{VMP}}$$

where ⊕ is the direct sum operator. Neurons in the same class generally innervate the same subunits in the neuropils, NO, IDFP, CCP, CVLP, and VMP, and hence can be described by identity matrices. However, a closer inspection revealed that the EIP class, which transmits signals into PB, exhibits a special pattern of innervation in IDFP by innervating one of the two subunits, DSB and VSB, alternatively (see Supplementary Presentation S1 for a detailed description of the innervation patterns). The PB LN class, which is the only PB interneuron class, also exhibits an innervation patterns that is different from other classes. Based on the observations, we constructed three global translation generators, one for PB input, one for PB output, and the other for PB interneuron classes. The only differences between the three generators lie in the two neuropils, PB and IDFP:

(9) $$\begin{array}{l} \text{ PB input}:\;T_{\text{in}}=\overset{\text{PB}}{\overset{⎴}{T^{\left(1,16\right)}}}\overset{\text{FB}}{\overset{⎴}{\underset{i\;=\;a,\ldots ,f}{⊕}T^{\left(1,8\right)}}}\overset{\text{EB}}{\overset{⎴}{\underset{i\;=\;A,O,C,P}{⊕}T^{\left(2,16\right)}}}⊕\overset{\text{NO}}{\overset{⎴}{I^{\left(8\right)}}}\\ \;⊕\overset{\text{IDFP}}{\overset{⎴}{T_{\text{IDFP}-\text{EIP}}}}⊕\overset{\text{CCP}}{\overset{⎴}{I^{\left(2\right)}}}⊕\overset{\text{CVLP}}{\overset{⎴}{I^{\left(2\right)}}}⊕\overset{\text{VMP}}{\overset{⎴}{I^{\left(4\right)}}}\\ \text{PB output}:\;T_{\text{out}}=\overset{\text{PB}}{\overset{⎴}{T^{\left(1,16\right)}}}\overset{\text{FB}}{\overset{⎴}{\underset{i\;=\;a,\ldots ,f}{⊕}T^{\left(1,8\right)}}}\overset{\text{EB}}{\overset{⎴}{\underset{i=A,O,C,P}{⊕}T^{\left(2,16\right)}}}⊕\overset{\text{NO}}{\overset{⎴}{I^{\left(8\right)}}}\\ \;⊕\overset{\text{IDFP}}{\overset{⎴}{I^{\left(10\right)}}}⊕\overset{\text{CCP}}{\overset{⎴}{I^{\left(2\right)}}}⊕\overset{\text{CVLP}}{\overset{⎴}{I^{\left(2\right)}}}⊕\overset{\text{VMP}}{\overset{⎴}{I^{\left(4\right)}}}\\ \text{PB inter}:\;T_{\text{inter}}=\overset{\text{PB}}{\overset{⎴}{T_{PB-PBLN}}}\overset{\text{FB}}{\overset{⎴}{\underset{i\;=\;a,\ldots ,f}{⊕}T^{\left(1,8\right)}}}\overset{\text{EB}}{\overset{⎴}{\underset{i\;=\;A,O,C,P}{⊕}T^{\left(2,16\right)}}}\\ \;⊕\overset{\text{NO}}{\overset{⎴}{I^{\left(8\right)}}}⊕\overset{\text{IDFP}}{\overset{⎴}{I^{\left(10\right)}}}⊕\overset{\text{CCP}}{\overset{⎴}{I^{\left(2\right)}}}⊕\overset{\text{CVLP}}{\overset{⎴}{I^{\left(2\right)}}}⊕\overset{\text{VMP}}{\overset{⎴}{I^{\left(4\right)}}} \end{array}$$

A detailed description of the translation generators in each neuropil is given in Table 1A and Supplementary Presentation S1 online.

**Table 1A:** **The translation generators required by each neuron class in each neuropil**.

**T**	**PB**	**FB**						**EB**				**NO**	**IDFP**	**CCP**	**CVLP**	**VMP**
		**a**	**b**	**c**	**d**	**e**	**f**	**A**	**O**	**C**	**P**					
PB LN	**T**_PB-PBLN_^*2^															
CIVP^*1^																
CVP	**T**^(1, 16)^													**I**^(2)^		**I**^(4)^
EIP	**T**^(1, 16)^								**T**^(2, 16)^	**T**^(2, 16)^	**T**^(2, 16)^		**T**_IDFP-EIP_^*2^			
PEI	**T**^(1, 16)^									**T**^(2, 16)^			**I**^(10)^			
PEN	**T**^(1, 16)^										**T**^(2, 16)^	**I**^(8)^				
PFN-F_*d*_N_2_	**T**^(1, 16)^				**T**^(1, 8)^							**I**^(8)^				
PFN-F_*e*_N_3_	**T**^(1, 16)^					**T**^(1, 8)^						**I**^(8)^				
PFN-F_*f*_N_4_	**T**^(1, 16)^						**T**^(1, 8)^					**I**^(8)^				
PFI-I_*RB*_	**T**^(1, 16)^			**T**^(1, 8)^	**T**^(1, 8)^								**I**^(10)^			
PFI-I_*HBl*_	**T**^(1, 16)^					**T**^(1, 8)^							**I**^(10)^			
PFI-I_*HBm*_	**T**^(1, 16)^			**T**^(1, 8)^	**T**^(1, 8)^	**T**^(1, 8)^	**T**^(1, 8)^						**I**^(10)^			
PFI-I_*L*+*R*−HBm_^*1^																

*1 *Neurons in CIVP and PFI-I_L+R−HBm_ classes can be fully described by mirror generators and do not require the translation generator*.

*2 *PB LN and EIP neurons require special generators in PB and IDFP, respectively. The special generators are described in Supplementary presentation S1*.

Similarly, we can also construct a global mirror generator to describe the relationship between each neuron and its contralateral counterpart.

(10) $$\begin{array}{l} M=M_{PB}⊕M_{FB}⊕M_{EB}⊕M_{NO}⊕M_{IDFP}⊕M_{CCP}⊕M_{CVLP}\\ \;⊕M_{VMP}\\ \;=\overset{\text{PB}}{\overset{⎴}{M^{\left(16\right)}}}\overset{\text{FB}}{\overset{⎴}{\underset{i\;=\;a,\ldots ,f}{⊕}M^{\left(8\right)}}}\overset{\text{EB}}{\overset{⎴}{\underset{i\;=\;A,O,C,P}{⊕}M^{\left(16\right)}}}⊕\overset{\text{NO}}{\overset{⎴}{M^{\left(2\right)}\times I^{\left(4\right)}}}\\ \;⊕\overset{\text{IDFP}}{\overset{⎴}{M^{\left(2\right)}\times I^{\left(5\right)}}}⊕\overset{\text{CCP}}{\overset{⎴}{M^{\left(2\right)}}}⊕\overset{\text{CVLP}}{\overset{⎴}{M^{\left(2\right)}}}⊕\overset{\text{VMP}}{\overset{⎴}{M^{\left(2\right)}\times I^{\left(2\right)}}} \end{array}$$

A detailed description of the mirror generators in each neuropil is given in Table 1B.

**Table 1B:** **The mirror generators used by each neuron class in each neuropil**.

**M**	**PB**	**FB**						**EB**				**NO**	**IDFP**	**CCP**	**CVLP**	**VMP**
		**a**	**b**	**c**	**d**	**e**	**f**	**A**	**O**	**C**	**P**					
PB LN	**M**^(16)^															
CIVP	**M**^(16)^														**M**^(2)^	
CVP	**M**^(16)^													**M**^(2)^		**M**^(2)^×**I**^(2)^
EIP	**M**^(16)^								**M**^(16)^	**M**^(16)^	**M**^(16)^		**M**^(2)^×**I**^(5)^			
PEI	**M**^(16)^									**M**^(16)^			**M**^(2)^×**I**^(5)^			
PEN	**M**^(16)^										**M**^(16)^	**M**^(2)^×**I**^(4)^				
PFN-F_*d*_N_2_	**M**^(16)^				**M**^(8)^							**M**^(2)^×**I**^(4)^				
PFN-F_*e*_N_3_	**M**^(16)^					**M**^(8)^						**M**^(2)^×**I**^(4)^				
PFN-F_*f*_N_4_	**M**^(16)^						**M**^(8)^					**M**^(2)^×**I**^(4)^				
PFI-**I**_RB_	**M**^(16)^			**M**^(8)^	**M**^(8)^								**M**^(2)^×**I**^(5)^			
PFI-**I**_HBl_	**M**^(16)^					**M**^(8)^							**M**^(2)^×**I**^(5)^			
PFI-**I**_HBm_	**M**^(16)^			**M**^(8)^	**M**^(8)^	**M**^(8)^	**M**^(8)^						**M**^(2)^×**I**^(5)^			
PFI-**I**_L+R−HBm_	**M**^(16)^			**M**^(8)^	**M**^(8)^	**M**^(8)^	**M**^(8)^									
Total	**M**^(16)^	**M**^(8)^	**M**^(8)^	**M**^(8)^	**M**^(8)^	**M**^(8)^	**M**^(8)^	**M**^(16)^	**M**^(16)^	**M**^(16)^	**M**^(16)^	**M**^(2)^×**I**^(4)^	**M**^(2)^×**I**^(5)^	**M**^(2)^	**M**^(2)^	**M**^(2)^×**I**^(2)^

Taking the PEN class for example, we can generate the PEN2 neuron type by applying **T** on PEN1 or **M** on PEN10:

(11) $$\begin{array}{l} T\times \text{PEN1}=\\ \left[\begin{array}{cccccccc} T_{PB} & \; & \; & \; & \; & \; & \; & \;\\ \; & T_{FB} & \; & \; & \; & \; & \; & \;\\ \; & \; & T_{EB} & \; & \; & \; & \; & \;\\ \; & \; & \; & I_{NO} & \; & \; & \; & \;\\ \; & \; & \; & \; & I_{IDFP} & \; & \; & \;\\ \; & \; & \; & \; & \; & I_{CCP} & \; & \;\\ \; & \; & \; & \; & \; & \; & I_{CVLP} & \;\\ \; & \; & \; & \; & \; & \; & \; & I_{VMP} \end{array}\right]\left[\begin{array}{c} PB_{R1}\\ FB_{0}\\ EB_{R8L8}\\ NO_{L1}\\ IDFP_{0}\\ CCP_{0}\\ CVLP_{0}\\ VMP_{0} \end{array}\right]\\ =\left[\begin{array}{c} T^{\left(1,16\right)}\times PB_{R1}\\ T^{\left(1,16\right)}\times FB_{0}\\ T^{\left(2,16\right)}\times EB_{R8L8}\\ I^{\left(8\right)}\times NO_{L1}\\ I^{\left(10\right)}\times IDFP_{0}\\ I^{\left(2\right)}\times CCP_{0}\\ I^{\left(2\right)}\times CVLP_{0}\\ I^{\left(4\right)}\times VMP_{0} \end{array}\right]=\left[\begin{array}{c} PB_{R2}\\ FB_{0}\\ EB_{L6L7}\\ NO_{L1}\\ IDFP_{0}\\ CCP_{0}\\ CVLP_{0}\\ VMP_{0} \end{array}\right]=\text{PEN2} \end{array}$$

or

(12) $$\begin{array}{l} M\times \text{PEN10}=\\ \text{​​}\left[\text{​}\begin{array}{cccccccc} M_{PB} & \; & \; & \; & \; & \; & \; & \;\\ \; & M_{FB} & \; & \; & \; & \; & \; & \;\\ \; & \; & M_{EB} & \; & \; & \; & \; & \;\\ \; & \; & \; & M_{NO} & \; & \; & \; & \;\\ \; & \; & \; & \; & M_{IDFP} & \; & \; & \;\\ \; & \; & \; & \; & \; & M_{CCP} & \; & \;\\ \; & \; & \; & \; & \; & \; & M_{CVLP} & \;\\ \; & \; & \; & \; & \; & \; & \; & M_{VMP} \end{array}\text{​​}\right]\text{​}\left[\text{​​}\begin{array}{c} PB_{L2}\\ FB_{0}\\ EB_{R6R7}\\ NO_{R1}\\ IDFP_{0}\\ CCP_{0}\\ CVLP_{0}\\ VMP_{0} \end{array}\text{​​}\right]\\ =\left[\begin{array}{c} M_{PB}\times PB_{L2}\\ M_{FB}\times FB_{0}\\ M_{EB}\times EB_{R6R7}\\ M_{NO}\times NO_{R1}\\ M_{IDFP}\times IDFP_{0}\\ M_{CCP}\times CCP_{0}\\ M_{CVLP}\times CVLP_{0}\\ M_{VMP}\times VMP_{0} \end{array}\right]=\left[\begin{array}{c} PB_{R2}\\ FB_{0}\\ EB_{L6l7}\\ NO_{L1}\\ IDFP_{0}\\ CCP_{0}\\ CVLP_{0}\\ VMP_{0} \end{array}\right]=\text{PEN2}. \end{array}$$

All PEN neurons on the ipsilateral side can be constructed by recursively applying the global translation generator on an initial neuron type (PEN1 or PEN16, Figure 2C). In addition, every PEN neuron can be constructed by applying the global mirror generator on the corresponding neuron on the contralateral side (Figure 2C):

(13) $$\begin{array}{l} \text{ PEN}2\;=T\times \text{PEN1}\\ \text{ PEN}3\;=T\times \text{PEN2}=T^{2}\times \text{PEN1}\\ \;\vdots \\ \text{ PEN}8\;=T\times \text{PEN7}=T^{7}\times \text{PEN1}\\ \text{ PEN}9\;=M\times \text{PEN1}\\ \text{PEN}10\;=M\times \text{PEN2}=M\times T\times \text{PEN1}\\ \;=T^{-1}\times \text{PEN9}=T^{T}\times \text{PEN9}=T^{T}\times M\times \text{PEN1}\\ \;\vdots \end{array}$$

In summary, except for a small number of atypical neuron types (described below), the innervation patterns of all PB-innervated neurons can be generated from 17 initial neuron types (three for the PB LN class, two for both the CVP and EIP classes, and one for each of the rest 10 classes) with four global generators (one mirror and three shift generators) (See Supplementary Presentation S1 online for a detailed description of each neuron class, the initial neurons, and the corresponding generators).

### Atypical innervation patterns

Some neurons do not obey the translation rule described by the global translation generators. We called these neurons “atypical.” Taking the PFN-F_*f*_N_4_ class for example, neurons that innervate adjacent units in PB also innervate the adjacent units in FB (Figure 2D). However, the type 2 and type 5 neurons in PFN-F_*f*_N_4_ class do not obey such a translation rule, and exhibit atypical innervation patterns in FB (Figure 2D). We investigated all atypical neurons and found that most of the atypical innervations can be classified into the two following patterns:

1. Splitting: In many neuron classes (PFN for example), each neuron innervates only one subunit in PB, FB, and NO. However, some neurons display different patterns from their peer neurons by innervating two subunits in PB or FB.
2. Standing: In PB/FB-innervating neuron classes, some neurons do not obey the translation rule and innervate the same subunits in PB or FB as the adjacent neuron types.

The splitting innervation pattern can be produced by a split generator, S, which is the sum of an identity matrix and a shift matrix. Taking the type 5 neurons in the PFN-F_*f*_N_4_ class for example, the FB component of this neuron type can be generated from the type 4 neurons by applying a translation generator and then a split generator:

$$\begin{array}{l} S_{A,\;FB}\times T_{FB}\times (\text{PFN}-{\text{F}}_{\text{f}}{\text{N}}_{4})4_{\text{FB}}=\\ \left[\begin{array}{cccccccc} 1 & 1 & \; & \; & \; & \; & \; & \;\\ \; & 1 & 1 & \; & \; & \; & \; & \;\\ \; & \; & 1 & 1 & \; & \; & \; & \;\\ \; & \; & \; & 1 & 1 & \; & \; & \;\\ \; & \; & \; & \; & 1 & 1 & \; & \;\\ \; & \; & \; & \; & \; & 1 & 1 & \;\\ \; & \; & \; & \; & \; & \; & 1 & 1\\ 1 & \; & \; & \; & \; & \; & \; & 1 \end{array}\right]\left[\begin{array}{cccccccc} \; & 1 & \; & \; & \; & \; & \; & \;\\ \; & \; & 1 & \; & \; & \; & \; & \;\\ \; & \; & \; & 1 & \; & \; & \; & \;\\ \; & \; & \; & \; & 1 & \; & \; & \;\\ \; & \; & \; & \; & \; & 1 & \; & \;\\ \; & \; & \; & \; & \; & \; & 1 & \;\\ \; & \; & \; & \; & \; & \; & \; & 1\\ 1 & \; & \; & \; & \; & \; & \; & \; \end{array}\right]\left[\begin{array}{c} 0\\ 0\\ 0\\ 0\\ 0\\ 2\\ 0\\ 0 \end{array}\right]=\\ \left[\begin{array}{c} 0\\ 0\\ 0\\ 2\\ 2\\ 0\\ 0\\ 0 \end{array}\right]=(\text{PFN}-{\text{F}}_{\text{f}}{\text{N}}_{4})5_{\text{FB}}^{.} \end{array}$$

The standing innervation pattern can be produced by a backward-translation generator, B, which is the inverse of the translation generator. Taking the FB component of the type 1 neurons of the PFN-F_*f*_N_4_ class again for example:

$$\begin{array}{l} B_{FB}\times S_{FB}\times (\text{PFN}-{\text{F}}_{\text{f}}{\text{N}}_{4})1_{\text{FB}}=\\ \left[\begin{array}{cccccccc} \; & \; & \; & \; & \; & \; & \; & 1\\ 1 & \; & \; & \; & \; & \; & \; & \;\\ \; & 1 & \; & \; & \; & \; & \; & \;\\ \; & \; & 1 & \; & \; & \; & \; & \;\\ \; & \; & \; & 1 & \; & \; & \; & \;\\ \; & \; & \; & \; & 1 & \; & \; & \;\\ \; & \; & \; & \; & \; & 1 & \; & \;\\ \; & \; & \; & \; & \; & \; & 1 & \; \end{array}\right]\left[\begin{array}{cccccccc} \; & 1 & \; & \; & \; & \; & \; & \;\\ \; & \; & 1 & \; & \; & \; & \; & \;\\ \; & \; & \; & 1 & \; & \; & \; & \;\\ \; & \; & \; & \; & 1 & \; & \; & \;\\ \; & \; & \; & \; & \; & 1 & \; & \;\\ \; & \; & \; & \; & \; & \; & 1 & \;\\ \; & \; & \; & \; & \; & \; & \; & 1\\ 1 & \; & \; & \; & \; & \; & \; & \; \end{array}\right]\left[\begin{array}{c} 0\\ 0\\ 0\\ 0\\ 0\\ 0\\ 2\\ 0 \end{array}\right]\;\\ =\left[\begin{array}{c} 0\\ 0\\ 0\\ 0\\ 0\\ 0\\ 2\\ 0 \end{array}\right]=(\text{PFN}-{\text{F}}_{\text{f}}{\text{N}}_{4})1_{\text{FB}} \end{array}$$

Since most atypical neurons can be described by the two special patterns, we can also construct the special generators for the atypical neurons (see Supplementary Presentation S1 online). Note that two of the atypical neurons (type 9 and type 18) in the EIP class cannot be described by either the splitting or standing innervation type. EIP neurons typically innervate three consecutive EB subunits with axonal terminals at the center and dendritic terminals on the sides. Instead, the two atypical EIP neurons innervate only one EB subunit with terminals of the mixed type (axon and dendrite).

### Impacts of the atypical neurons on the network structure

The existence of the atypical neurons is particularly interesting. We argue that the atypical neurons likely characterize special functional requirements in addition to the general functions formed by the “typical” neurons (see Discussion). To further investigate this issue, we studied how atypical neurons alter the properties of the central complex network. To this end, we compared several commonly used measures of network structures between the “observed” and the “model” networks. The “observed network” is the one described in Lin C.-Y. et al. (2013), (as summarized in the Supplementary Table S1), while the “model network” was created solely by applying the global generators (Equations 9, 10) to the initial neurons in each class. Therefore, the main difference between the model and observed networks lies in the existence of the atypical innervation patterns. The observed network consists of 194 neuron types and the modeled network consists of 172 neuron types. Out of the 194 neuron types in the observed network, 46 are atypical neuron types (see Supplementary Figure S3 online) and the rest 148 are typical types which are shared between the observed and model networks. The detailed description of the observed and the modeled networks is given in the Supplementary Presentation S1 online.

We first analyzed several basic network properties (Rubinov and Sporns, 2010; Kaiser, 2011), including characteristic path length (Watts and Strogatz, 1998), global efficiency (Watts and Strogatz, 1998; Latora and Marchiori, 2001), clustering coefficient (Holland and Leinhardt, 1971; Watts and Strogatz, 1998), modularity (Newman, 2004), and small-worldness (Humphries and Gurney, 2008). We discovered that except for the smaller clustering coefficient in the model network, the two networks are very similar with each other (Table 2).

**Table 2:** **Network properties of the observed and model networks in comparison with the random and ***C. Elegans*** networks**.

	**Characteristic path length**	**Global efficiency**	**Clustering coefficient**	**Modularity**	**Small-worldness**
Observed Network	3.2068	0.1433	0.0563	0.4130 ± 0.0128	1.4687 ± 0.1097
Model Network	3.3021	0.1409	0.0479	0.4194 ± 0.0020	1.4061 ± 0.1220

*The values of modularity and small-worldness were calculated based on 1,000 trials and are presented as mean ± standard deviation*.

**Figure 3:**
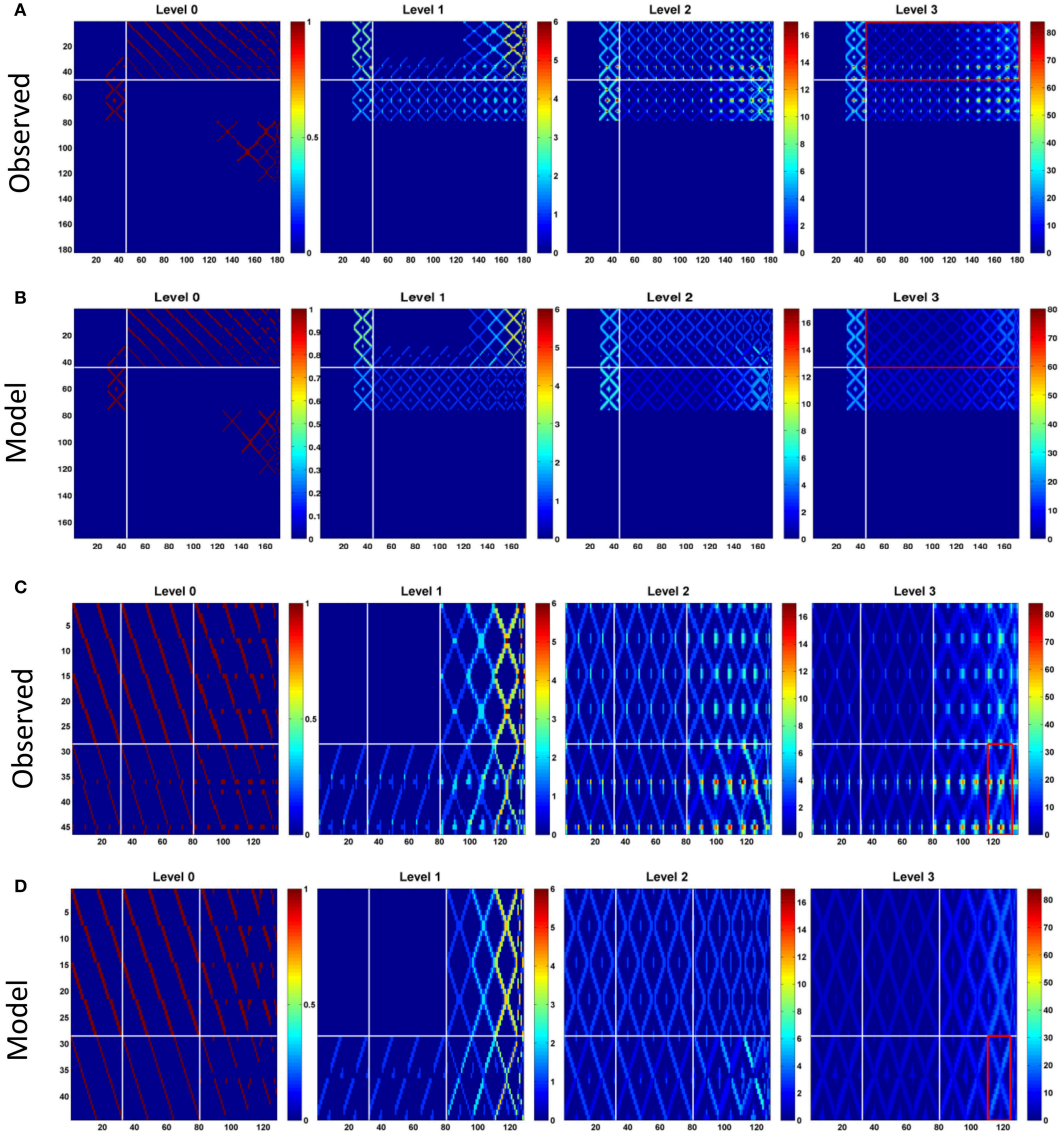
**Connection matrices at different propagation levels for, (A)**. The observed network and, **(B)**. the model network. The vertical axis represents the index of the source neuron types while the horizontal axis is for the destination neuron types. The order of the neuron type index follows that in Supplementary Table S1 but with local neurons (PB LN) removed. So there are only 184 neuron types presented in the matrices. Each element in the matrices indicates the number of paths (represented by the color) connecting the source and destination neuron types at the given propagation levels. In general, the number of paths increases with the level for both networks as expected. However, the difference between the two networks increases dramatically at the higher levels. The observed network has large maximum path numbers, which is more than twice of that in the model network at the propagation level 3. The white lines separate the PB input neurons (before the lines) from the PB output neurons (after the lines). The red rectangles outline the portion of the matrices shown in **(C)** for the observed and **(D)** for the model networks. In **(C**,**D)**, horizontal white lines separate two classes of input neurons (from top to down: CVP and EIP) and the vertical white lines separate output neuron classes (from left to right: PEI, PEN, PFN, and PFI). The red rectangle marks the region formed by EIP->PFI-I_*HBI*_ classes, where the largest path number in the observed network is located.

The network structure analysis, based on the conventional network features, is not sensitive to the presence/absence of the atypical neurons. We hypothesize that although the global structural properties examined in the foregoing analysis may not be altered by the presence of atypical neurons, they are still likely to change certain network properties which can be revealed by analyzing information propagation in the network. To this end, we examined the connection matrices at high propagation levels (Lin et al., 2014) (see Section Methods). We discovered that while the connection matrices of the observed and model networks are very similar at the low propagation levels (levels 0 and 1), the difference between the two networks dramatically increases at the high levels (level 2 and above) (Figures 3A,B). To elaborate upon the differences between the connection matrices of the two networks, we further examined the portion of the matrices formed by PB input and output neurons (Figures 3C,D). This portion of the matrices shows how signals entering PB from the input neurons travel to the output neurons. The input neurons comprise the EIP and CVP classes, while the output neurons comprise 9 neuron classes including PEN, PEI, PFN (three classes), and PFI (four classes). For the observed network, several highly connected neuron pairs emerge at the propagation level 3 with path numbers up to 80, characterizing information hotspots of the network. In contrast, no hotspot is observed in the model network at the same level.

**Figure 4:**
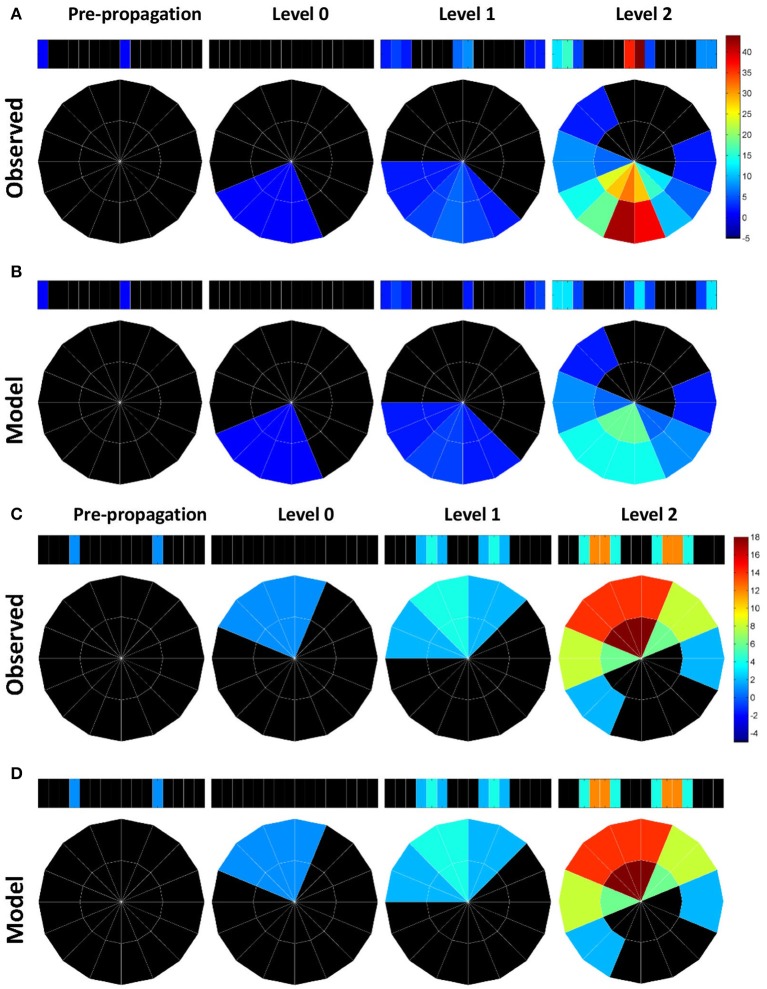
**Information propagation in the EB-PB feedback loop. (A)** If starting from the subunits R1 and L8 of PB (the first panel from the left) in the observed network, a signal quickly propagates to the central and lateral subunits in PB via EB after two levels of propagation. Colors indicate the number of hits by the signal in each subunit. **(B)** In the model network, the same starting subunit leads to a much smaller hit number. **(C)** If we start from L5 and R4 subunits in PB, the signal propagates between L5, R4, and neighboring PB subunits via EB. **(D)** Same as in c but for the model network. The model network exhibits the same propagation pattern and number of hits as those in the observed network.

The influence of the atypical neurons on the path numbers is interesting, but what is the impact on information propagation and what does it mean to circuit functions? Large path numbers at the high levels have been shown to be the result of feedback (recurrent) connections (Lin et al., 2014). Given that the input neuron class EIP receives strong feedback from the output neuron classes PEN and PEI in EB (Figure 1B), we conjectured that the increase of the path numbers in the observed network mainly results from the atypical neuron-dependent strengthening of the feedback circuits between EB and PB. To test this conjecture, we traced how a signal propagates in the circuit with and without the atypical neurons. We first selected one subunit in PB and started from neurons with dendrites innervating this subunit. By assuming that a signal can propagate from a neuron to all its downstream neurons, we traced the signal starting from a selected PB subunit for several levels of synaptic transmissions and counted the number of hits in each subunit. A hit in a subunit is defined as an input, or the arrival of a signal, from one neuron. We repeated the process for each PB subunit and discovered that while starting from some PB subunits yields minor or no difference between the observed and model networks, starting from the medial (PB L1 and PB R1) and from the most lateral (PB L8 and R8) subunits produces a distinct number of hits between the two networks (Figure 4). The observed network exhibits a large number of hits in the PB medial (R1 and L1) subunits as well as in some EB (R8 and L8) subunits, while the model network does not. The result suggests that the existence of the atypical neurons strengthens the recurrent circuit between specific regions of PB and EB. This observation is particularly interesting when we consider some of the known functions of the central complex (see Discussion).

**Figure 5:**
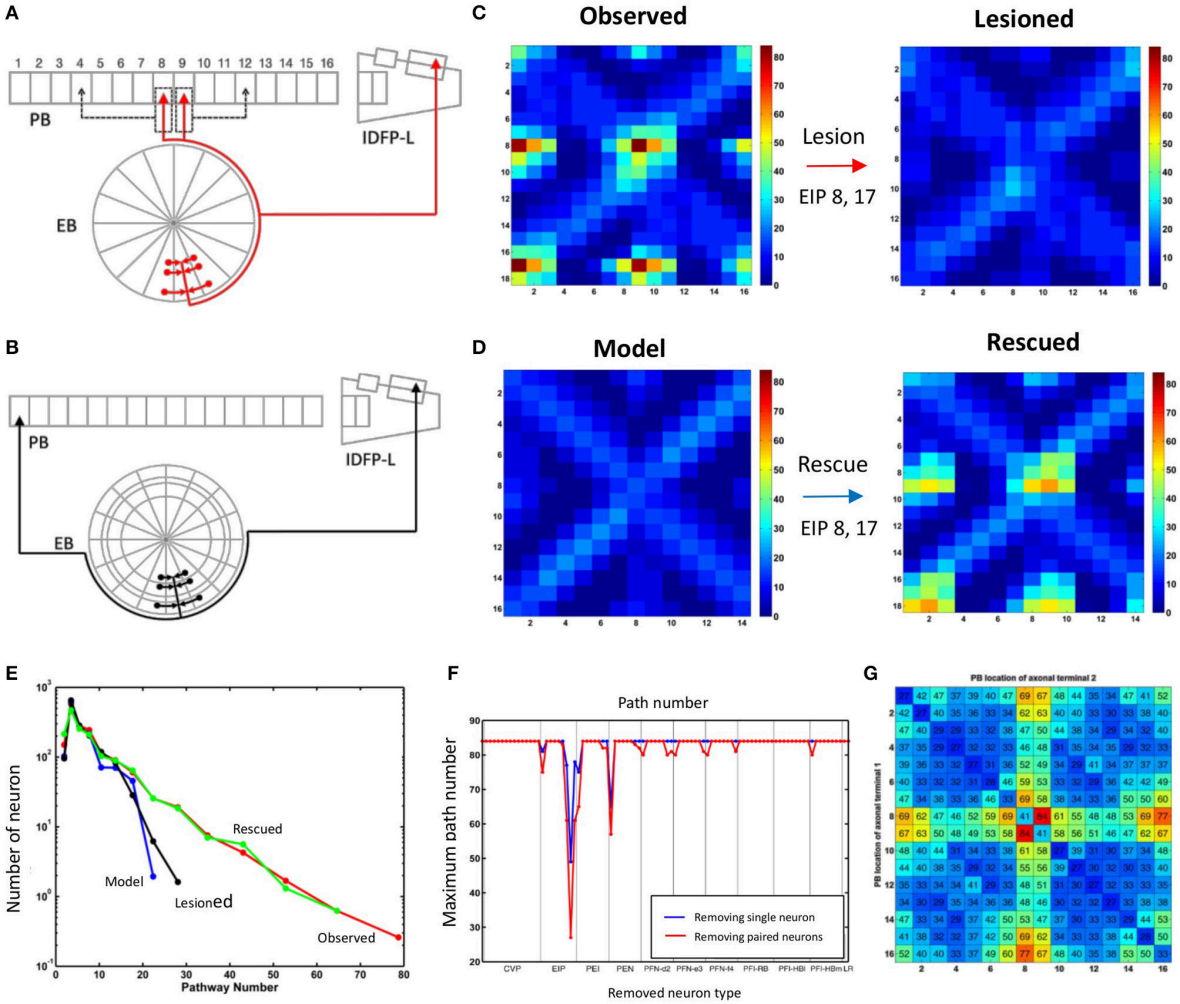
**The unique impact of the atypical neurons EIP8 and EIP17 on the central complex network. (A)** The EIP8 neuron type in the observed network innervates the medial PB subunits. **(B)** In contrast, the predicted EIP8 in the model network innervates the lateral PB subunits. The EIP17 neuron is a mirroring of EIP8 in both networks. **(C)** Removing EIP8 and EIP17 from the observed network completely abolishes the information hotspots at the propagation level 3. **(D)** Interestingly, the hotspots can be partially “rescued” at the level 3 in the model network by adding the atypical neurons EIP8 and EIP17 back. In order to visualize the detailed changes, here we only show a small portion (corresponds to the red rectangle in Figures 3C,D) of the connection matrix. **(E)** The distribution of path numbers at the propagation level 3 between every pair of neurons for observed, model, lesioned, and rescued networks. The distributions for the observed and rescued networks both exhibit a long tail, characterizing the existence of neuron pairs with high path numbers. **(F)** The effects of different neuron types on the maximum path number at the propagation level 3. By removing a single neuron type (blue) or a neuron type together with its contralateral counterpart (red) one at a time from the observed network, we discovered that only EIP8 and EIP17 cause the most significant reduction in the maximum path number, more than any other neuron types. **(G)** The locations of the axonal terminals are crucial for the effect of EIP8 and EIP17 on the path number. By randomly allocating the two axonal terminals of EIP8 in PB and keeping the EIP17 neuron symmetric to EIP8, we observed that only in their native terminal locations, EIP8 and EIP17 neurons lead to the highest maximum path numbers. The vertical and the horizontal axes indicate the positions of the axonal terminals in PB (index of PB subunit). The number in each small square labels the maximum path number of the network at the level 3 with the indicated terminal positions. The diagonal represents the condition in which the EIP8 and EIP17 neurons only innervate one single PB subunit.

With further examination, we discovered that the difference between the two networks in terms of the maximum path number (as shown in Figure 3) and the pattern of information propagation (as shown in Figure 4) are mainly caused by a pair of atypical EIP neuron types (EIP8 and EIP17) (Figures 5A,B). The influence of the EIP8 and EIP17 in the path number can be demonstrated by performing the “lesion” and “rescue” tests (Figures 5C,D). By removing (mimicking the lesion experiment in neurophysiological studies) only the two neuron types, EIP 8 and EIP 17, from the observed network, we found that the information hotspots no longer present in the high-level connection matrices (Figure 5C). Moreover, we could partially rescue the hotspots in the model network by only adding the two atypical neuron type back to it (Figure 5D).

In addition to the impact of EIP8 and EIP17 on the hotspots as shown in Figure 5, the impact of the two neuron types on the entire network can be better seen by examining the distributions of the path numbers of all neuron pairs of the networks. We found that with the two atypical neuron types (in observed and rescued networks), the distribution of the path number exhibits a long tail (Figure 5E). In contrast, without the two neuron types (in the model or in the lesioned network), the networks exhibit much narrower and short-tailed distributions. We further investigated whether any other neuron type produces a similar impact on the network in terms of the path numbers. To this end, we removed an arbitrary neuron type and its contralateral counterpart from the observed network and calculated its maximum path number at the propagation level 3. We found that while removal of some pairs of neuron types reduced the maximum path number with various degrees, the removal of EIP8 & EIP17 led to the most severe reduction of the maximum path number (from 80 to 26, Figure 5F).

The impact of the two atypical neuron types, EIP8 and EIP17, on the information propagation is evident based on the present analyses. We next asked why the atypical innervation patterns of these neuron types cause such an impact on information propagation. Compared to other typical EIP neuron types, EIP8 and EIP17 have two unique features in their innervation patterns: (1) their axons project to two subunits instead of only one as do other EIP neurons, and (2) their axons project to PB L1 and PB L2, instead of to PB L8 or PB R8 as predicted by the generators. To evaluate how the impact of the two neurons may originate from their unique features, we recorded the maximum path number in the input-output matrix (as shown in Figures 3C,D) at the propagation level 3 while relocating the axonal terminals of EIP8 to arbitrary PB subunits (as shown by dashed arrows in Figure 5A). We kept EIP17's projections symmetric to those of relocated EIP8 axons. We found that the two neuron types yield the largest maximum path number with their original innervation pattern and all axonal terminal relocations led to smaller maximum path numbers (Figure 5G). Interestingly, the innervation pattern predicted by the generators (corresponds to the upper right corner of the matrix) yields the smallest path numbers among all possible configurations. The functional implication of the unique innervation patterns of the two atypical neuron types is discussed in the Discussion.

## Discussion

In the present study, we demonstrated a novel approach in neural circuit analysis for the central complex. We showed that the innervation patterns of the PB-innervating neurons can be largely described (or predicted) by only a few generators with a small set of initial neuron types, except for the atypical ones. We investigated the atypical neurons and found that they greatly enhance the recurrent connections in the network. This difference is mainly contributed by a pair of atypical neuron types, EIP8 and EIP17, which exhibit a unique innervation pattern that propagates signals between the medial and lateral PB subunits via EB.

**Figure 6:**
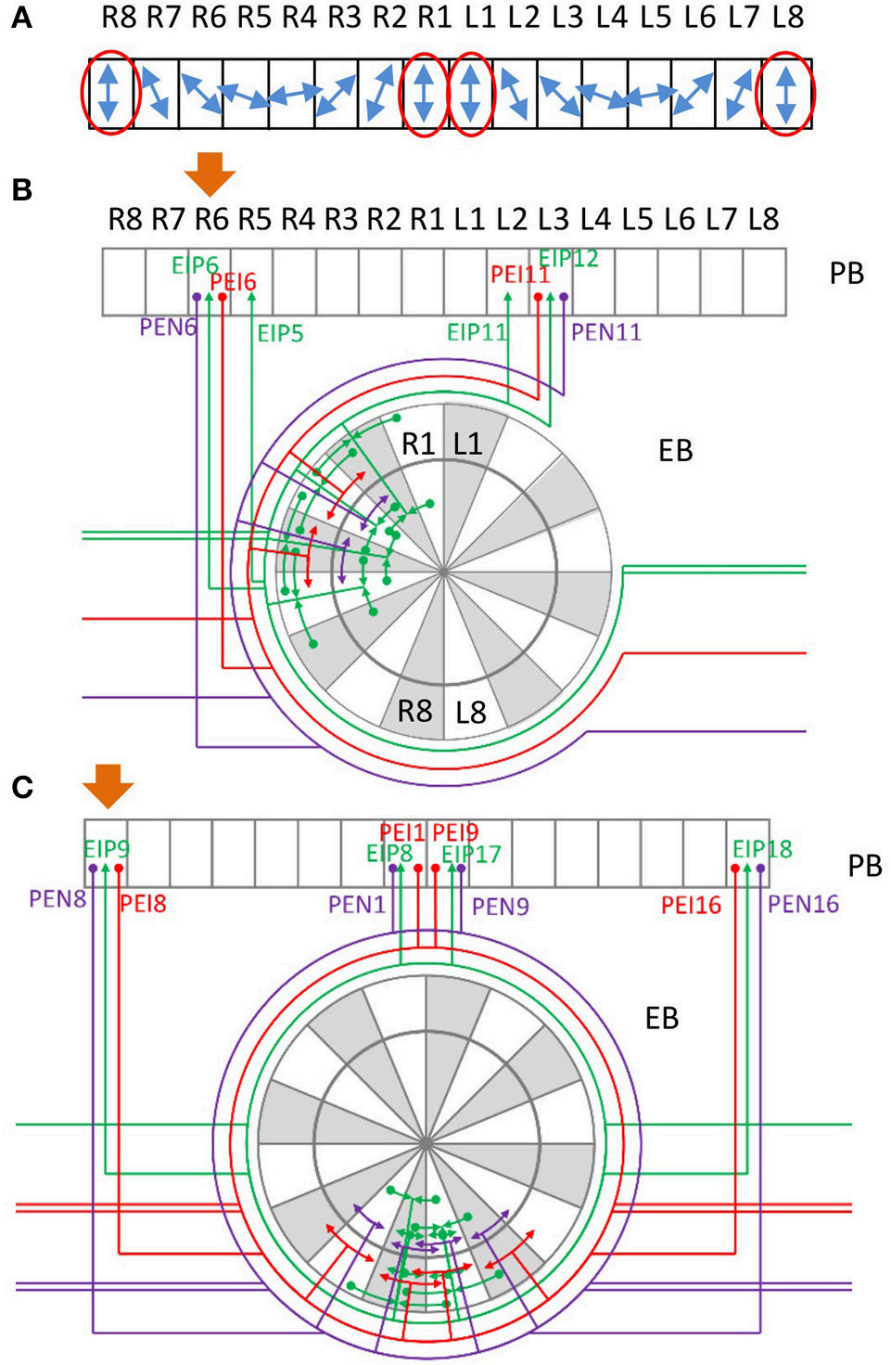
**Possible role of the neurons EIP 8 and 17 in integrating sensory information. (A)** A schematic of E-vector selectivity in PB. The blue arrows indicate the orientation of E-vector of polarized light selected by each of the 16 PB subunits observed in locust (Heinze and Homberg, 2007, 2009; Heinze et al., 2009; Homberg et al., 2011). Each polarization direction is selected by two contralateral subunits except for the vertical direction, which is selected by two lateral and two medial subunits (red ovals). **(B)** Example of recurrent circuits between PB and EB. Assuming that a signal starts from PB R6, as indicated by the orange arrow, a strong recurrent signal propagation is quickly established between PB R6, PB L3, and several EB subunits. Note that in locust, R6 and L3 are selective to the same polarized light direction. **(C)** A special recurrent circuit involving the atypical neurons EIP8 and EIP17. Assuming PB R8 as the starting subunit of a signal (orange arrow), it propagates to EB R8 and L8, and then quickly reaches the medial PB subunits R1 and L1. In a few steps, a strong recurrent signal propagation is established between PB R8, R1, L1, L8, and several EB subunits. Note that in locust, all of these four PB subunits are selective to the vertically polarized light.

Does the unique innervation pattern of EIP8 and EIP17 carry any specific function that is distinct from other typical neurons? Although detailed neural functional experiments are required in order to answer the question, some hints may be obtained by considering a number of earlier studies, which revealed the role of locust PB in encoding polarization of lights (Heinze and Homberg, 2007; Heinze et al., 2009; Homberg et al., 2011). According to these studies, the E-vector of polarized light, represented in an angular coordinate system, is encoded in PB, which has a linear structure (Figure 6A). The E-vector angle encoded by each PB subunit is represented in a mirroring fashion between the left and right sides of PB. Under such a mapping, each E-vector angle is encoded by two subunits, one from each side of PB. Therefore, the information processed by the two sides of PB, in particularly by the subunits that encode the same E-vector angle, has to be exchanged to some degree in order to maintain the integrity of spatial perception. Indeed, this is what the recurrent connections between PB and EB offer (Figure 6B). Each PEN or PEI neuron sends information from PB to EB, in which EIP neurons feed the information back to the PB subunits which encode the same E-vector angle in both sides. However, the situation becomes more complicated when we consider the vertical E-vector, which is encoded by four subunits at both ends of each side of PB (Figure 6A, red ovals). The circuits formed by the PEN/PEI/EIP would not be capable of offering the connection between the four subunits if EIP 8 and EIP17 follow the “typical” pattern as predicted by the generators. By projecting to the two medial subunits instead of the predicted lateral subunits, the atypical EIP8 and EIP17 provide strong connections between the four subunits that encode the vertical E-vector (Figure 6C). We stress that it is not clear whether PB-innervating neurons in *Drosophila* encode polarization of lights like those in locusts do. Nevertheless, the unique innervation pattern of the atypical neurons EIP8 and EIP17 may suggest a role in maintaining the integrity of sensory information. This speculation requires rigorous tests in future experimental studies.

One may ask, if the two atypical neuron types EIP8 and EIP17 are so crucial to the network structure, wouldn't loss of them cause a catastrophic damage to the whole network? We suggest that this is not the case if each neuron type consists of multiple neurons as redundancy. An anatomical study has reported the existence of isomorphic neurons in several neuron types in the central complex (Young and Armstrong, 2010b). Whether the EIP class also possesses isomorphic neurons for each of its neuron types remains to be verified experimentally.

Beside the atypical neurons, the discovery of a simple rule (the generators) that can be used to produce the major part of the circuit is also significant. Intuitively, one would think that the connectivity of the central complex network should be described by a host of connection rules because the network consists of more than a dozen neuron classes which exhibit different projection patterns across several neuropils. To our knowledge, this is the first demonstration that the connectome of a brain region can be reduced to a set of deterministic equations at the single neuron level. From the development point of view, it is a cost-saving strategy if the genetic system only needs to encode the rule for circuit construction and the innervation patterns of the initial neurons instead of encoding the pattern of every neuron.

A recent study on the central complex neuroanatomy of *Drosophila* suggested an updated circuit (Wolff et al., 2015). In the study, the authors identified two medial PB subunits, which were previously not identified. They also suggested an updated segmentation of PB which contains 9 layers instead of 6 layers, as described in Lin C.-Y. et al. (2013). However, most neuron classes described in Lin C.-Y. et al. (2013) are identified in Wolff et al. (2015). The general innervation patterns for most neuron classes were also confirmed by Wolff et al. (2015), except for the EIP type, which is claimed to have only dendritic domains presented in EB. These new findings do not go against the idea of neural network generators proposed in the present study. However, since the Wolff et al. (2015) did not provide detailed type-by-type innervation patterns for all neuron classes such as Lin C.-Y. et al. (2013) did, it is difficult to fully update the proposed mathematical description of the central complex network at the current stage. But based on the data provided in Wolff et al. (2015), their updated circuit can be easily incorporated into our system by adding new elements to the innervation vectors and modifying the initial neurons.

In the present study, we identified the significance of specific network nodes, i.e., atypical neurons, by the propagation level analysis. The analysis is very effective for neural networks (Lin et al., 2014). Typical network analyses focus on the level 0 propagation (direct connections). However, neural networks are highly recurrent, and theoretical studies have suggested that some functions are associated with strong interactions between neurons in recurrent circuits (Wang, 1999, 2002; Brunel, 2000; Laje and Buonomano, 2013). Therefore, by calculating the number of paths at the high propagation levels, which characterize recurrent circuits, we may be able to gain insights into the functional significance of specific neurons which do not seem to be special when only looking at their direct connections. Taking working memory for example, a prevalent theory suggests that working memory is encoded in persistent neural activity supported by strong local feedback excitation and global inhibition (Compte et al., 2000; Constantinidis and Wang, 2004). Indeed, the three neuron classes, EIP, PEN, and PEI, form local excitation between individual EB and PB subunits, while the EB ring neurons (not described in the present study) (Hanesch et al., 1989), which innervate all subunits in individual EB rings, may play roles in global inhibition. Interestingly, this argument is consistent with a recent experimental study in which persistent activity bumps that encode spatial orientation memory were observed in EB (Seelig and Jayaraman, 2013, 2015). The matrix generator approach described here greatly helps us with the construction of a computational model in a follow-up study which reproduces some of the observations reported in Seelig and Jayaraman (2015). However, we caution that one should not assess the functions of a neural circuit only based on the anatomical data because static connections do not provide sufficient information about functions due to the dynamical nature of neurons and synapses (Seung, 2011; Alivisatos et al., 2012; Bargmann and Marder, 2013). Any theoretical study on neural circuit functions should be conducted based on experimental observations from both anatomical and functional studies.

The matrix representation presented in this study was constructed by visual inspection and this is relatively straightforward for a small and structured network such as the central complex. To generalize our mathematical approach for other larger neural networks, we are developing a software tool to extract various connectivity patterns from arbitrary networks based on graph theory and statistics. The tool will help people to inspect whether there are hidden and statistically significant connectivity patterns embedded in a neural network that is visually complex and random.

It is interesting that our analysis on network properties revealed small clustering coefficient and small-worldness for both observed and model networks, indicating that they are not well fit by the classic small-world model. The result is not so surprising considering recent reports which suggested that the neural networks may not be so “small” as commonly noted and the small-worldness of a neural network is influenced by the techniques used to acquire and analyze the data (Muller et al., 2014; Hilgetag and Goulas, 2016).

In the present study, we did not identify the impact of other atypical neurons on the signal propagation. This is because those atypical neurons are part of the one-way circuit from PB to FN or from FN to IDFP, and the direct feedback circuit from FN to PB or EB is not yet identified. We believe that once the detailed description of the recurrent circuit between FN and PB or EB is fully available, one can also discover the importance of these PFN and PFI atypical neurons by using the same analysis presented here.

There are a few limitations of the mathematical approaches introduced in the present study. The generation matrices provide a very concise way to describe the structure of a network. However, this approach is not suitable for networks that do not have clear topographical organization. The propagation level analysis helps to locate the sub-circuits that are potentially interesting from the theoretical perspective. However, the approach cannot identify their exact functions.

In conclusion, the present study is significant in several aspects: (1) it demonstrates how a neural circuit can be largely constructed from simple mathematical rules, which suggests the simplicity in the design principle behind complicated neural circuits, (2) it provides an effective measure (the path numbers at the high propagation levels) for evaluating the impact of single neurons on the signal propagation, and, (3) it shows that the atypical neurons play crucial roles in routing information propagation in the central complex.

## Author contributions

CS and CL designed the study. PC and CL wrote the main manuscript text. PC and TS prepared figures. All authors reviewed the manuscript.

## Funding

The work was supported by Ministry of Science and Technology grants 105-2633-B-007-001, 105-2112-M-029-002, and 105-2311-B-007-012-MY3, and by the Aim for the Top University Project of the Ministry of Education.

### Conflict of interest statement

The authors declare that the research was conducted in the absence of any commercial or financial relationships that could be construed as a potential conflict of interest.
