# Supplementary material for: The Topographical Mapping in Drosophila Central Complex Network and Its Signal Routing
Source: Front Neuroinform. 2017 Apr 10;11:26. doi: 10.3389/fninf.2017.00026 (PMC5385387; doi:10.3389/fninf.2017.00026)
Supplement: Supplementary file 1 [file Table1.PDF]

Supplementary Material

Complex Network from Simple Rules – The Topographical Mapping in Drosophila Central Complex Network and its Signal Routing

Po-Yen Chang[#1], Ta-Shun Su[#1], Chi-Tin Shih[*2,3], and Chung-Chuan Lo[*1,4]

[#] **These authors contributed equally**

*** Correspondence:**

Chung-Chuan Lo: cclo@mx.nthu.edu.tw

Chi-Tin Shih: shih.chi.tin@gmail.com

Supplementary Material Table

Table S1. Neuron types and naming. Comparison between the simplified version used in the present study and the original version used in Lin et al 2013 [1].

Simplified Version		Original Version				
**Class**	**Type**	**No.**	**Superclass**	**Class**	**Type**	**Sample**
PB LN	1	1	PB LN	PB LN	PB$_{\text{R3-8 glomeruli; axon: R7,R8}}$	7
	2	2			PB$_{\text{L3-8 glomeruli; axon: L7,L8}}$	7
	3	3			PB$_{\text{R1-8,L1-7 glomeruli; axon: R6,L1}}$	12
	4	4			PB$_{\text{R1–8,L1-7 glomeruli; axon: R5,L2}}$	12
	5	5			PB$_{\text{R1-8,L1-7 glomeruli; axon: R4,L3}}$	23
	6	6			PB$_{\text{R1-7,L1-8 glomeruli; axon: R3,L4}}$	15
	7	7			PB$_{\text{R1-7,L1-8 glomeruli; axon: R2,L5}}$	12
	8	8			PB$_{\text{R1-7,L1-8 glomeruli; axon: R1,L6}}$	10
	9	9			PB$_{\text{R1-8,L1-8 glomeruli; axon: R8,R1,L7}}$	13
	10	10			PB$_{\text{R1-8,L1-8 glomeruli; axon: R7,L1,L8}}$	23
CIVP	1	11	CIVP	$C_LI_LV_LP$	CVLP$_{\text{L-medial}}$–IDFP$_{\text{L-HB-lateral}}$–VMP$_{\text{L-lateral}}$→PB$_{\text{R1-8,L1-8}}$	3
	2	12		$C_RI_RV_RP$	CVLP$_{\text{R-medial}}$–IDFP$_{\text{R-HB-lateral}}$–VMP$_{\text{R-lateral}}$→PB$_{\text{R1-8,L1-8}}$	--
CVP	1	13	CVP	C_LV_LP	CCP$_{\text{L-ventral}}$–VMP$_{\text{L-dorsal}}$→PB$_{\text{R1,R2}}$	--
	2	14			CCP$_{\text{L-ventral}}$–VMP$_{\text{L-dorsal}}$→PB$_{\text{R2,R3}}$	1
	3	15			CCP$_{\text{L-ventral}}$–VMP$_{\text{L-dorsal}}$→PB$_{\text{R3,R4}}$	--
	4	16			CCP$_{\text{L-ventral}}$–VMP$_{\text{L-dorsal}}$→PB$_{\text{R4,R5}}$	--
	5	17			CCP$_{\text{L-ventral}}$–VMP$_{\text{L-dorsal}}$→PB$_{\text{R5,R6}}$	--
	6	18			CCP$_{\text{L-ventral}}$–VMP$_{\text{L-dorsal}}$→PB$_{\text{R6,R7}}$	--
	7	19			CCP$_{\text{L-ventral}}$–VMP$_{\text{L-dorsal}}$→PB$_{\text{R7,R8}}$	--
	8	20			CCP$_{\text{L-ventral}}$–VMP$_{\text{L-dorsal}}$→PB$_{\text{L1,L2}}$	1
	9	21			CCP$_{\text{L-ventral}}$–VMP$_{\text{L-dorsal}}$→PB$_{\text{L2,L3}}$	--
	10	22			CCP$_{\text{L-ventral}}$–VMP$_{\text{L-dorsal}}$→PB$_{\text{L3,L4}}$	2
	11	23			CCP$_{\text{L-ventral}}$–VMP$_{\text{L-dorsal}}$→PB$_{\text{L4,L5}}$	--
	12	24			CCP$_{\text{L-ventral}}$–VMP$_{\text{L-dorsal}}$→PB$_{\text{L5,L6}}$	--
	13	25			CCP$_{\text{L-ventral}}$–VMP$_{\text{L-dorsal}}$→PB$_{\text{L6,L7}}$	--
	14	26			CCP$_{\text{L-ventral}}$–VMP$_{\text{L-dorsal}}$→PB$_{\text{L7,L8}}$	1
	15	27		C_RV_RP	CCP$_{\text{R-ventral}}$–VMP$_{\text{R-dorsal}}$→PB$_{\text{R1,R2}}$	--
	16	28			CCP$_{\text{R-ventral}}$–VMP$_{\text{R-dorsal}}$→PB$_{\text{R2,R3}}$	1
VP	17	29	CVP	C_RV_RP	CCP$_{\text{R-ventral}}$–VMP$_{\text{R-dorsal}}$→PB$_{\text{R3,R4}}$	--
	18	30			CCP$_{\text{R-ventral}}$–VMP$_{\text{R-dorsal}}$→PB$_{\text{R4,R5}}$	1
	19	31			CCP$_{\text{R-ventral}}$–VMP$_{\text{R-dorsal}}$→PB$_{\text{R5,R6}}$	--
	20	32			CCP$_{\text{R-ventral}}$–VMP$_{\text{R-dorsal}}$→PB$_{\text{R6,R7}}$	1

	21	33			CCP$_{R\text{-ventral}}$–VMP$_{R\text{-dorsal}}$→PB$_{R7,R8}$	2
	22	34			CCP$_{R\text{-ventral}}$–VMP$_{R\text{-dorsal}}$→PB$_{L1,L2}$	--
	23	35			CCP$_{R\text{-ventral}}$–VMP$_{R\text{-dorsal}}$→PB$_{L2,L3}$	--
	24	36			CCP$_{R\text{-ventral}}$–VMP$_{R\text{-dorsal}}$→PB$_{L3,L4}$	--
	25	37			CCP$_{R\text{-ventral}}$–VMP$_{R\text{-dorsal}}$→PB$_{L4,L5}$	--
	26	38			CCP$_{R\text{-ventral}}$–VMP$_{R\text{-dorsal}}$→PB$_{L5,L6}$	--
	27	39			CCP$_{R\text{-ventral}}$–VMP$_{R\text{-dorsal}}$→PB$_{L6,L7}$	--
	28	40			CCP$_{R\text{-ventral}}$–VMP$_{R\text{-dorsal}}$→PB$_{L7,L8}$	--
	1	45			EB$_{L5C,O,P;\ L7C,O,P}$→EB$_{L6C,O,P}$–IDFP$_{L\text{-VSB}}$–PB$_{R1}$	1
	2	42			EB$_{L3C,O,P;\ L5C,O,P}$→EB$_{L4C,O,P}$–IDFP$_{L\text{-DSB}}$–PB$_{R2}$	3
	3	46			EB$_{L1C,O,P;\ L3C,O,P}$→EB$_{L2C,O,P}$–IDFP$_{L\text{-VSB}}$–PB$_{R3}$	--
	4	43			EB$_{L1C,O,P;\ R2C,O,P}$→EB$_{R1C,O,P}$–IDFP$_{L\text{-DSB}}$–PB$_{R4}$	5
	5	47		EI$_L$P	EB$_{R2C,O,P;\ R4C,O,P}$→EB$_{R3C,O,P}$–IDFP$_{L\text{-VSB}}$–PB$_{R5}$	4
	6	44			EB$_{R4C,O,P;\ R6C,O,P}$→EB$_{R5C,O,P}$–IDFP$_{L\text{-DSB}}$–PB$_{R6}$	2
	7	48			EB$_{R6C,O,P;\ R8C,O,P}$→EB$_{R7C,O,P}$–IDFP$_{L\text{-VSB}}$–PB$_{R7}$	11
	8	41			EB$_{L7C,O,P;\ R8C,O,P}$→EB$_{L8C,O,P}$–IDFP$_{L\text{-DSB}}$–PB$_{R1,L1}$	4
EIP	9	49	EIP		EB$_{R8C,P}$→EB$_{R8C,P}$–IDFP$_{L\text{-DSB}}$–PB$_{R8}$	5
	10	54			EB$_{R5C,O,P;\ R7C,O,P}$→EB$_{R6C,O,P}$–IDFP$_{R\text{-VSB}}$–PB$_{L1}$	1
	11	51			EB$_{R3C,O,P;\ R5C,O,P}$→EB$_{R4C,O,P}$–IDFP$_{R\text{-DSB}}$–PB$_{L2}$	3
	12	55			EB$_{R1C,O,P;\ R3C,O,P}$→EB$_{R2C,O,P}$–IDFP$_{R\text{-VSB}}$–PB$_{L3}$	4
	13	52			EB$_{R1C,O,P;\ L2C,O,P}$→EB$_{L1C,O,P}$–IDFP$_{R\text{-DSB}}$–PB$_{L4}$	6
	14	56		EI$_R$P	EB$_{L2C,O,P;\ L4C,O,P}$→EB$_{L3C,O,P}$–IDFP$_{R\text{-VSB}}$–PB$_{L5}$	3
	15	53			EB$_{L4C,O,P;\ L6C,O,P}$→EB$_{L5C,O,P}$–IDFP$_{R\text{-DSB}}$–PB$_{L6}$	5
	16	57			EB$_{L6C,O,P;\ L8C,O,P}$→EB$_{L7C,O,P}$–IDFP$_{R\text{-VSB}}$–PB$_{L7}$	4
	17	50			EB$_{R7C,O,P;\ L8C,O,P}$→EB$_{R8C,O,P}$–IDFP$_{R\text{-DSB}}$–PB$_{L1,R1}$	1
	18	58			EB$_{L8C,P}$→EB$_{L8C,P}$–IDFP$_{R\text{-DSB}}$–PB$_{L8}$	3
	1	59			PB$_{R1}$→EB$_{L7C;\ L6C}$–IDFP$_{L\text{-DSB}}$	1
	2	60			PB$_{R2}$→EB$_{L5C;\ L4C}$–IDFP$_{L\text{-DSB}}$	--
	3	61			PB$_{R3}$→EB$_{L3C;\ L2C}$–IDFP$_{L\text{-DSB}}$	--
PEI	4	62	PEI	PEI$_L$	PB$_{R4}$→EB$_{L1C;\ R1C}$–IDFP$_{L\text{-DSB}}$	--
	5	63			PB$_{R5}$→EB$_{R2C;\ R3C}$–IDFP$_{L\text{-DSB}}$	--
	6	64			PB$_{R6}$→EB$_{R4C;\ R5C}$–IDFP$_{L\text{-DSB}}$	1
	7	65			PB$_{R7}$→EB$_{R6C;\ R7C}$–IDFP$_{L\text{-DSB}}$	--
	8	66		PEI$_L$	PB$_{R8}$→EB$_{R8C;\ L8C}$–IDFP$_{L\text{-DSB}}$	--
	9	67			PB$_{L1}$→EB$_{R7C;\ R6C}$–IDFP$_{R\text{-DSB}}$	--
PEI	10	68	PEI		PB$_{L2}$→EB$_{R5C;\ R4C}$–IDFP$_{R\text{-DSB}}$	--
	11	69		PEI$_R$	PB$_{L3}$→EB$_{R3C;\ R2C}$–IDFP$_{R\text{-DSB}}$	--
	12	70			PB$_{L4}$→EB$_{R1C;\ L1C}$–IDFP$_{R\text{-DSB}}$	1
	13	71			PB$_{L5}$→EB$_{L2C;\ L3C}$–IDFP$_{R\text{-DSB}}$	1

	14	72			$PB_{L6}\rightarrow EB_{L4C;\ L5C}\text{—}IDFP_{R\text{-}DSB}$	1
	15	73			$PB_{L7}\rightarrow EB_{L6C;\ L7C}\text{—}IDFP_{R\text{-}DSB}$	1
	16	74			$PB_{L8}\rightarrow EB_{L8C;\ R8C}\text{—}IDP_{R\text{-}DSB}$	3
	1	75			$PB_{R1}\rightarrow EB_{R8P;\ L8P}\text{—}NO_{L1}$	1
	2	76			$PB_{R2}\rightarrow EB_{L7P;\ L6P}\text{—}NO_{L1}$	--
	3	77			$PB_{R3}\rightarrow EB_{L5P;\ L4P}\text{—}NO_{L1}$	3
	4	78		PEN_L	$PB_{R4}\rightarrow EB_{L3P;\ L2P}\text{—}NO_{L1}$	--
	5	79			$PB_{R5}\rightarrow EB_{L1P;\ R1P}\text{—}NO_{L1}$	2
	6	80			$PB_{R6}\rightarrow EB_{R2P;\ R3P}\text{—}NO_{L1}$	1
	7	81			$PB_{R7}\rightarrow EB_{R4P;\ R5P}\text{—}NO_{L1}$	--
PEN	8	82	PEN		$PB_{R8}\rightarrow EB_{R6P;\ R7P}\text{—}NO_{L1}$	--
	9	83			$PB_{L1}\rightarrow EB_{R8P;\ L8P}\text{—}NO_{R1}$	1
	10	84			$PB_{L2}\rightarrow EB_{R6P;\ R7P}\text{—}NO_{R1}$	1
	11	85			$PB_{L3}\rightarrow EB_{R4P;\ R5P}\text{—}NO_{R1}$	--
	12	86		PEN_R	$PB_{L4}\rightarrow EB_{R2P;\ R3P}\text{—}NO_{R1}$	--
	13	87			$PB_{L5}\rightarrow EB_{L1P;\ R1P}\text{—}NO_{R1}$	1
	14	88			$PB_{L6}\rightarrow EB_{L3P;\ L2P}\text{—}NO_{R1}$	--
	15	89			$PB_{L7}\rightarrow EB_{L5P;\ L4P}\text{—}NO_{R1}$	1
	16	90			$PB_{L8}\rightarrow EB_{L7P;\ L6P}\text{—}NO_{R1}$	--
	1	91			$PB_{R1}\rightarrow FB_{L4d}\text{—}NO_{L2}$	2
	2	92			$PB_{R2}\rightarrow FB_{L4d}\text{—}NO_{L2}$	--
	3	93			$PB_{R3}\rightarrow FB_{L3d}\text{—}NO_{L2}$	2
	4	94		PFN_L	$PB_{R4}\rightarrow FB_{L2d}\text{—}NO_{L2}$	1
	5	95			$PB_{R5}\rightarrow FB_{L1d,\ R1d}\text{—}NO_{L2}$	1
$PFN\text{-}F_dN_2$	6	96	PFN		$PB_{R6}\rightarrow FB_{R2d}\text{—}NO_{L2}$	--
	7	97			$PB_{R7}\rightarrow FB_{R3d}\text{—}NO_{L2}$	--
	8	98			$PB_{R8}\rightarrow FB_{R4d}\text{—}NO_{L2}$	--
	9	115			$PB_{L1}\rightarrow FB_{R4d}\text{—}NO_{R2}$	1
	10	116		PFN_R	$PB_{L2}\rightarrow FB_{R4d}\text{—}NO_{R2}$	1
	11	117			$PB_{L3}\rightarrow FB_{R3d}\text{—}NO_{R2}$	1
	12	118			$PB_{L4}\rightarrow FB_{R2d}\text{—}NO_{R2}$	--
	13	119			$PB_{L5}\rightarrow FB_{R1d,L1d}\text{—}NO_{R2}$	1
$PFN\text{-}F_dN_2$	14	120		PFN_R	$PB_{L6}\rightarrow FB_{L2d}\text{—}NO_{R2}$	--
	15	121			$PB_{L7}\rightarrow FB_{L3d}\text{—}NO_{R2}$	--
	16	122	PFN		$PB_{L8}\rightarrow FB_{L4d}\text{—}NO_{R2}$	--
	1	99			$PB_{R1}\rightarrow FB_{L4e}\text{—}NO_{L3}$	--
$PFN\text{-}F_eN_3$	2	100		PFN_L	$PB_{R2}\rightarrow FB_{L4e}\text{—}NO_{L3}$	1
	3	101			$PB_{R3}\rightarrow FB_{L3e}\text{—}NO_{L3}$	1

	4	102			$PB_{R4} \rightarrow FB_{L2e}-NO_{L3}$	1
	5	103			$PB_{R5} \rightarrow FB_{L1e,R1e}-NO_{L3}$	1
	6	104			$PB_{R6} \rightarrow FB_{R2e}-NO_{L3}$	--
	7	105			$PB_{R7} \rightarrow FB_{R3e}-NO_{L3}$	1
	8	106			$PB_{R8} \rightarrow FB_{R4e}-NO_{L3}$	--
	9	123			$PB_{L1} \rightarrow FB_{R4e}-NO_{R3}$	--
	10	124			$PB_{L2} \rightarrow FB_{R4e}-NO_{R3}$	1
	11	125			$PB_{L3} \rightarrow FB_{R3e}-NO_{R3}$	1
	12	126		PFN_R	$PB_{L4} \rightarrow FB_{R2e}-NO_{R3}$	--
	13	127			$PB_{L5} \rightarrow FB_{R1e,L1e}-NO_{R3}$	--
	14	128			$PB_{L6} \rightarrow FB_{L2e}-NO_{R3}$	1
	15	129			$PB_{L7} \rightarrow FB_{L3e}-NO_{R3}$	1
	16	130			$PB_{L8} \rightarrow FB_{L4e}-NO_{R3}$	1
	1	107			$PB_{R1} \rightarrow FB_{L4f}-No_{L4}$	--
	2	108			$PB_{R2} \rightarrow FB_{L4f}-No_{L4}$	1
	3	109			$PB_{R3} \rightarrow FB_{L3f}-No_{L4}$	5
	4	110		PFN_L	$PB_{R4} \rightarrow FB_{L2f}-No_{L4}$	2
	5	111			$PB_{R5} \rightarrow FB_{L1f,R1f}-No_{L4}$	4
	6	112			$PB_{R6} \rightarrow FB_{R2f}-No_{L4}$	--
	7	113			$PB_{R7} \rightarrow FB_{R3f}-No_{L4}$	4
$PFN-F_fN_4$	8	114			$PB_{R8} \rightarrow FB_{R4f}-No_{L4}$	4
	9	131			$PB_{L1} \rightarrow FB_{R4f}-NO_{R4}$	2
	10	132			$PB_{L2} \rightarrow FB_{R4f}-NO_{R4}$	1
	11	133			$PB_{L3} \rightarrow FB_{R3f}-NO_{R4}$	4
	12	134		PFN_R	$PB_{L4} \rightarrow FB_{R2f}-NO_{R4}$	2
	13	135			$PB_{L5} \rightarrow FB_{R1f,L1f}-NO_{R4}$	6
	14	136			$PB_{L6} \rightarrow FB_{L2f}-NO_{R4}$	1
	15	137			$PB_{L7} \rightarrow FB_{L3f}-NO_{R4}$	3
	16	138			$PB_{L8} \rightarrow FB_{L4f}-NO_{R4}$	4
$PFI-I_{RB}$	1	139	PFI	PFI_L	$PB_{L1}-FB_{L4c,d} \rightarrow IDFP_{L-RB}$	1
	2	140			$PB_{R1}-FB_{L4c,d} \rightarrow IDFP_{L-RB}$	1
	3	141			$PB_{R2}-FB_{L3c,d} \rightarrow IDFP_{L-RB}$	2
	4	142			$PB_{R3}-FB_{L2c,d} \rightarrow IDFP_{L-RB}$	1
	5	143		PFI_L	$PB_{R4}-FB_{L1c,d} \rightarrow IDFP_{L-RB}$	1
$PFI-I_{RB}$	6	144	PFI		$PB_{R4}-FB_{R1c,d} \rightarrow IDFP_{L-RB}$	5
	7	145			$PB_{R5}-FB_{R2c,d} \rightarrow IDFP_{L-RB}$	1
	8	145			$PB_{R6}-FB_{R3c,d} \rightarrow IDFP_{L-RB}$	2
	9	147			$PB_{R7}-FB_{R4c,d} \rightarrow IDFP_{L-RB}$	2
	10	165			$PB_{R1}-FB_{R4c,d} \rightarrow IDFP_{R-RB}$	2

	11	166		PFI_R	PB_{L1}–$FB_{R4c,d}$→$IDFP_{R-RB}$	3
	12	167			PB_{L2}–$FB_{R3c,d}$→$IDFP_{R-RB}$	4
	13	168			PB_{L3}–$FB_{R2c,d}$→$IDFP_{R-RB}$	3
	14	169			PB_{L4}–$FB_{R1c,d}$→$IDFP_{R-RB}$	1
	15	170			PB_{L4}–$FB_{L1c,d}$→$IDFP_{R-RB}$	5
	16	171			PB_{L5}–$FB_{L2c,d}$→$IDFP_{R-RB}$	1
	17	172			PB_{L6}–$FB_{L3c,d}$→$IDFP_{R-RB}$	2
	18	173			PB_{L7}–$FB_{L4c,d}$→$IDFP_{R-RB}$	--
PFI-I_{HBl}	1	149		PFI_L	PB_{L1}–$FB_{L3e,L4e}$→$IDFP_{L-HB-lateral}$	4
	2	148			$PB_{L1,R1}$–$FB_{L3e,L4e}$→$IDFP_{L-HB-lateral}$	25
	3	150			PB_{R1}–$FB_{L2e,L3e}$→$IDFP_{L-HB-lateral}$	24
	4	151			PB_{R2}–$FB_{L1e,L2e}$→$IDFP_{L-HB-lateral}$	3
	5	152			PB_{R3}–$FB_{R1e,L1e}$→$IDFP_{L-HB-lateral}$	2
	6	153			PB_{R4}–$FB_{R2e,R1e}$→$IDFP_{L-HB-lateral}$	--
	7	154			PB_{R5}–$FB_{R3e,R2e}$→$IDFP_{L-HB-lateral}$	--
	8	155			PB_{R6}–$FB_{R4e,R3e}$→$IDFP_{L-HB-lateral}$	25
	9	156		PFI_R	PB_{R7}–$FB_{R4e,R3e}$→$IDFP_{L-HB-lateral}$	--
	10	175			PB_{R1}–$FB_{R3e,R4e}$→$IDFP_{R-HB-lateral}$	--
	11	174			$PB_{R1,L1}$–$FB_{R3e,R4e}$→$IDFP_{R-HB-lateral}$	23
	12	176			PB_{L1}–$FB_{R2e,R3e}$→$IDFP_{R-HB-lateral}$	35
	13	177			PB_{L2}–$FB_{R1e,R2e}$→$IDFP_{R-HB-lateral}$	2
	14	178			PB_{L3}–$FB_{L1e,R1e}$→$IDFP_{R-HB-lateral}$	2
	15	179			PB_{L4}–$FB_{L2e,L1e}$→$IDFP_{R-HB-lateral}$	3
	16	180			PB_{L5}–$FB_{L3e,L2e}$→$IDFP_{R-HB-lateral}$	1
	17	181			PB_{L6}–$FB_{L4e,L3e}$→$IDFP_{R-HB-lateral}$	28
	18	182			PB_{L7}–$FB_{L4e,L3e}$→$IDFP_{R-HB-lateral}$	1
PFI-I_{HBm}	1	158		PFI_L	PB_{L1}–$FB_{L4c,d,e,f; L3c,d}$→$IDFP_{L-HB-medial}$	3
	2	157			$PB_{R1,L1}$–$FB_{L3c,d,e,f; L2c,d}$→$IDFP_{L-HB-medial}$	1
PFI-I_{HBm}	3	159	PFI	PFI_L	PB_{R1}–$FB_{L2c,d,e,f; L1c,d}$→$IDFP_{L-HB-medial}$	2
	4	160			PB_{R2}–$FB_{L1c,d,e,f; R1c,d}$→$IDFP_{L-HB-medial}$	4
	5	161			PB_{R3}–$FB_{R1c,d,e,f; R2c,d}$→$IDFP_{L-HB-medial}$	2
	6	162			PB_{R4}–$FB_{R2c,d,e,f; R3c,d}$→$IDFP_{L-HB-medial}$	1
	7	163			PB_{R5}–$FB_{R3c,d,e,f; R4c,d}$→$IDFP_{L-HB-medial}$	6
	8	164			PB_{R6}–$FB_{R4c,d,e,f; R3c,d}$→$IDFP_{L-HB-medial}$	34
	9	184		PFI_R	PB_{L1}–$FB_{R3c,d; R4c,d,e,f}$→$IDFP_{R-HB-medial}$	--
	10	183			$PB_{L1,R1}$–$FB_{R2c,d; R3c,d,e,f}$→$IDFP_{R-HB-medial}$	--
	11	185			PB_{R1}–$FB_{R1c,d; R2c,d,e,f}$→$IDFP_{R-HB-medial}$	2
	12	186			PB_{R2}–$FB_{L1c,d; R1c,d,e,f}$→$IDFP_{R-HB-medial}$	4

	13	187			PB_{R3}–$FB_{L2c,d; L1c,d,e,f}$→$IDFP_{R-HB-medial}$	--
	14	188			PB_{R4}–$FB_{L3c,d; L2c,d,e,f}$→$IDFP_{R-HB-medial}$	3
	15	189			PB_{R5}–$FB_{L4c,d; L3c,d,e,f}$→$IDFP_{R-HB-medial}$	2
	16	190			PB_{R6}–$FB_{L3c,d; L4c,d,e,f}$→$IDFP_{R-HB-medial}$	18
$PFI\text{-}I_{L+R-HBm}$	1	191		PFI_{L+R}	PB_{R1}–$FB_{R1c,d,e,f; R2c,d}$→$IDFP_{L+R-HB-medial}$	2
	2	192			PB_{R3}–$FB_{R4c,d,e,f; R3c,d}$→$IDFP_{L+R-HB-medial}$	14
	3	193			PB_{L1}–$FB_{L1c,d,e,f; L2c,d}$→$IDFP_{L+R-HB-medial}$	6
	4	194			PB_{L3}–$FB_{L4c,d,e,f; L3c,d}$→$IDFP_{L+R-HB-medial}$	22

Reference:

1. Lin C-Y, Chuang C-C, Hua T-E, Chen C-C, Dickson BJ, Greenspan RJ, et al. A Comprehensive Wiring Diagram of the Protocerebral Bridge for Visual Information Processing in the Drosophila Brain. Cell Reports. 2013;3: 1739–1753. doi:10.1016/j.celrep.2013.04.022
