# Supplementary material for: The Topographical Mapping in Drosophila Central Complex Network and Its Signal Routing
Source: Front Neuroinform. 2017 Apr 10;11:26. doi: 10.3389/fninf.2017.00026 (PMC5385387; doi:10.3389/fninf.2017.00026)
Supplement: Supplementary file 6 [file Presentation1.PDF]

Supplementary Material

Complex Network from Simple Rules – The Topographical Mapping in Drosophila Central Complex Network and its Signal Routing

Po-Yen Chang[#1], Ta-Shun Su[#1], Chi-Tin Shih[*2,3], and Chung-Chuan Lo[*1,4]

[#] These authors contributed equally

*** Correspondence:**

Chung-Chuan Lo: cclo@mx.nthu.edu.tw

Chi-Tin Shih: shih.chi.tin@gmail.com

Supplementary Material Presentation

Presentation S1

Innervation pattern for each neuron class and the corresponding generators

1. PB LN class

   The PB LN class consists of all local neurons of PB. The class can be further divided into three subclasses, 6G, 15G and 16G (G stands for glomeruli), based on the number of innervated subunits (or glomeruli) (Fig A). Both 6G and 16G subclasses contain only one pair of neuron type and can be described just by the reflection rule. The 15G subclass contains three pairs of neuron type and has to be described by the translation and reflection rules. The PB LN class requires three initial neurons, one for each subclass. Because only innervating 15 subunits, the translation generator T for the15G subclass takes special forms:

$$T_{\text{PB-PBLN}} = \begin{bmatrix}
& 1 &&&&&&&&&&&&&& \\
&& 1 &&&&&&&&&&&&& \\
&&& 1 &&&&&&&&&&&& \\
&&&& 1 &&&&&&&&&&& \\
&&&&& 1 &&&&&&&&&& \\
&&&&&& 1 &&&&&&&&& \\
&&&&&&& 1 &&&&&&&& \\
&&&&&&&& 1 &&&&&&& \\
&&&&&&&&& 1 &&&&&& \\
&&&&&&&&&& 1 &&&&& \\
&&&&&&&&&&& 1 &&&& \\
&&&&&&&&&&&& 1 &&& \\
&&&&&&&&&&&&& 1 && \\
&&&&&&&&&&&&&& 1 & \\
1 &&&&&&&&&&&&&&& \\
&&&&&&&&&&&&&&&
\end{bmatrix}$$

   for the types 3-5 and

$$T_{\text{PB-PBLN}} = \begin{bmatrix}
&&&&&&&&&&&&&&& \\
&& 1 &&&&&&&&&&&&& \\
&&& 1 &&&&&&&&&&&& \\
&&&& 1 &&&&&&&&&&& \\
&&&&& 1 &&&&&&&&&& \\
&&&&&& 1 &&&&&&&&& \\
&&&&&&& 1 &&&&&&&& \\
&&&&&&&& 1 &&&&&&& \\
&&&&&&&&& 1 &&&&&& \\
&&&&&&&&&& 1 &&&&& \\
&&&&&&&&&&& 1 &&&& \\
&&&&&&&&&&&& 1 &&& \\
&&&&&&&&&&&&& 1 && \\
&&&&&&&&&&&&&& 1 & \\
&&&&&&&&&&&&&&& 1 \\
& 1 &&&&&&&&&&&&&&
\end{bmatrix}$$

   for the types 6-8.

(A)

PB LN Class

Neuron type index	PB R8	R7	R6	R5	R4	R3	R2	R1	L1	L2	L3	L4	L5	L6	L7	L8	*1
1	2	2	1	1	1	1											6G
2											1	1	1	1	2	2	
3	1	1	2	1	1	1	1	1	2	1	1	1	1	1	1		15G
4	1	1	1	2	1	1	1	1	1	2	1	1	1	1	1		
5	1	1	1	1	2	1	1	1	1	1	2	1	1	1	1		
6		1	1	1	1	2	1	1	1	1	1	2	1	1	1	1	
7		1	1	1	1	1	2	1	1	1	1	1	2	1	1	1	
8		1	1	1	1	1	1	2	1	1	1	1	1	2	1	1	
9	2	1	1	1	1	1	1	2	1	1	1	1	1	1	2	1	16G
10	1	2	1	1	1	1	1	1	2	1	1	1	1	1	1	2	

(B)

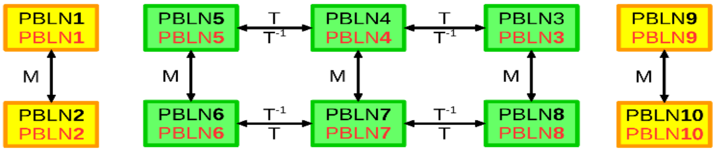

Figure A. (A) The innervation table of the PB LN class. Each row indicates the innervation pattern of a neuron type. Cells labeled as 1 represent the dendritic innervation while 2's represent axonal ones. The class can be divided into three subclasses (6G, 15G and 16G) based on the number of innervated subunits. (B) The generator diagram. Each rectangle represent an unique neuron type and each arrow indicates a generator operation, with the generators above denoting the rightward and the generators below denoting the leftward operations. The neuron types labeled in black are for the model network while the labels in red are for the observed network. There is no atypical neuron in the class. So both observed and model networks share the same neuron types. The class requires three initial neurons, types 1, 5 and 9, for the subclasses 6G, 15G and 16G, respectively.

*1: we only list neuropils that are innervated by the described neuron class. Same in all following figures.

2. CIVP class

The CIVP neurons transmit signals from VMP, CVLP and IDFP to PB. Only one type of neuron in the CIVP class was observed. Lin et al. 2013 predicted a second type of neurons which is the mirror of the first one. The CIVP neurons innervate all PB subunits, but only one subunit in IDFP, CVLP and VMP (Figure B).

(A)

CIVP Class

Neuron type *2	PB R8	R7	R6	R5	R4	R3	R2	R1	L1	L2	L3	L4	L5	L6	L7	L8
1	2	2	2	2	2	2	2	2	2	2	2	2	2	2	2	2
2	2	2	2	2	2	2	2	2	2	2	2	2	2	2	2	2

Neuron type *2	IDFP HBm-R	HBl-R	DSB-R	VSB-R	RB-R	HBm-L	HBl-L	DSB-L	VSB-L	RB-L	CVLP R	CVLP L	VMP d-R	l-R	d-L	l-L
1							1					1				1
2		1									1			1		

(B)

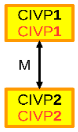

Figure B. (A) The innervation table and (B) the generator diagram of the CIVP class. The neurons type index labeled in purple indicates the type that was predicted in Lin et al. 2013.

3. CVP class

The CVP neurons transmit signals from CCP and VMP to PB. The class contains 28 neuron types (Figure C). The 28 neuron types can be further divided into two subclasses. The first subclass includes types 1-7 and 22-28 and the second includes types 8-14 and 15-21. Note that only type 2 neurons in the first subclass was observed and the remaining types are all predicted (labeled in purple) (Lin et al., 2013). In the second subclass 7 neuron types were observed. The CVP class requires two initial neurons, one for each subclass.

(A)

CVP Class

Neuron type index

	PB																CCP		VMP			
	R8	R7	R6	R5	R4	R3	R2	R1	L1	L2	L3	L4	L5	L6	L7	L8	R	L	d-R	l-R	d-L	l-L
1							2	2										1			1	
2						2	2											1			1	
3					2	2												1			1	
4				2	2													1			1	
5			2	2														1			1	
6		2	2															1			1	
7	2	2																1			1	
8									2	2								1			1	
9										2	2							1			1	
10											2	2						1			1	
11												2	2					1			1	
12													2	2				1			1	
13														2	2			1			1	
14															2	2		1			1	
15							2	2									1		1			
16						2	2										1		1			
17					2	2											1		1			
18				2	2												1		1			
19			2	2													1		1			
20		2	2														1		1			
21	2	2															1		1			
22									2	2							1		1			
23										2	2						1		1			
24											2	2					1		1			
25												2	2				1		1			
26													2	2			1		1			
27														2	2		1		1			
28															2	2	1		1			

(B)

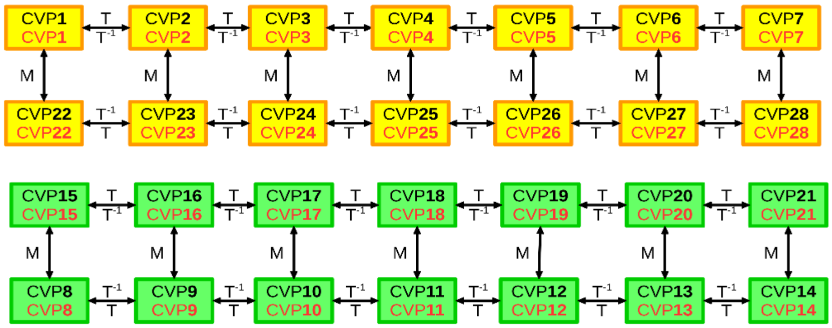

Figure C. (A) The innervation table and (B) the generator diagrams for the CVP class. The class can be divided into two subclasses with CVP1 and CVP15 as the initial neurons for the first and the second subclasses, respectively. Note that most neuron types in the class are predicted.

4. EIP class

The EIP neurons transmit signals from EB to PB, IDFP and (back to) EB. Most neuron types in EIP class only innervate one PB and one IDFP subunits, but innervate three consecutive subunits (with the axonal terminal in the middle subunit and the dendritic terminals on the sides) in C, O, and P rings in EB. Note that when the terminal of a neuron type in PB shifts one subunit with respected to the adjacent neuron type, the terminals in EB shift two subunits (Figure D). The EIP class has two pairs of atypical neurons and requires two initial neurons for the observed network but only one initial neuron for the model network. Unlike other neuron classes (PFI's for example) which innervate the same IDFP subunit for all ipsilateral neuron types, EIP neurons switch between two subunits (DSB and VSB) alternatively across ipsilateral neuron types. Hence, we need a local translation generator to describe the innervation of EIP neurons in IDFP. The generator takes the following form:

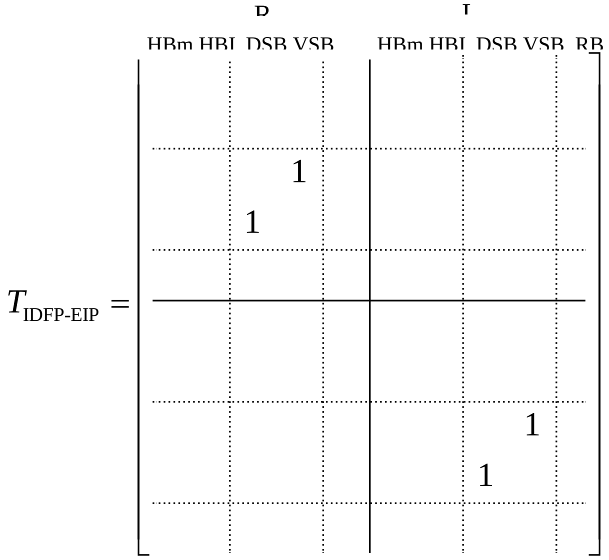

The generator shifts innervation in VSB to DSB, or vice versa for both sides (R and L).

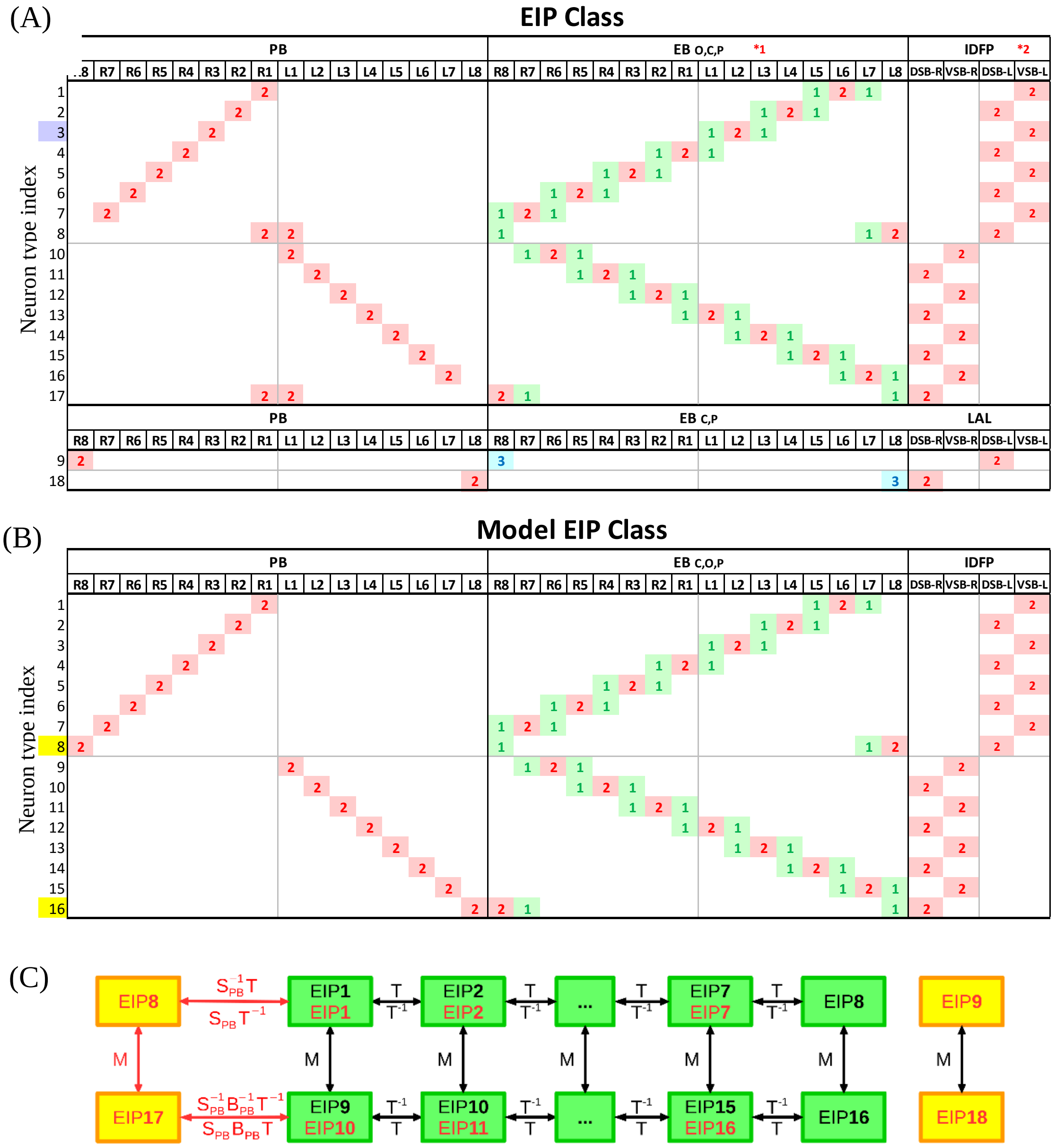

Figure D. The innervation table of the EIP class neurons in (A) the observed and (B) the model network. (C) The generator diagram. The EIP class has two pairs of atypical neurons: 1) the types 8 and 17 which innervate two subunits in PB, and 2) the types 9 and 18 which extend both of axonal and dendritic terminals into one subunit in only two rings (C and P) of EB. The type 8 neuron can be generated from the type 1 by a backward shift (T^{-1}) followed by a split in PB (S_{PB}). The types 9 and 18 are removed in the model network. The EIP class requires two initial neurons (EIP7 and EIP9) for the observed network but requires only one initial neuron (EIP8) for the model network.

*1. The subscripts C, O and P indicate that the neurons innervate the same subunits in all three EB rings (C, O and P). *2. IDFP consists of 10 subunits, but here we only list DSB and VSB subunits in both sides. *3. Neuron types generated by the generators but not observed are labeled in yellow in (B).

5. PEI class

The PEI neurons transmit signals from PB to EB (rings C) and IDFP with a very regular innervation pattern (Figure E). No atypical neuron exists in this class. Only one initial neuron is required for generating the entire class of neurons.

PEI Class

(A)

Neuron type index	PB																EB c																IDFP	
	R8	R7	R6	R5	R4	R3	R2	R1	L1	L2	L3	L4	L5	L6	L7	L8	R8	R7	R6	R5	R4	R3	R2	R1	L1	L2	L3	L4	L5	L6	L7	L8	DSB-R	DSB-L
1								1																						2	2			2
2							1																					2	2					2
3						1																				2	2							2
4					1																			2	2									2
5				1																		2	2											2
6			1																	2	2													2
7		1																2	2															2
8	1																2															2		2
9									1									2	2														2	
10										1										2	2												2	
11											1											2	2										2	
12												1												2	2								2	
13													1													2	2						2	
14														1														2	2				2	
15															1															2	2		2	
16																1	2															2	2	

(B)

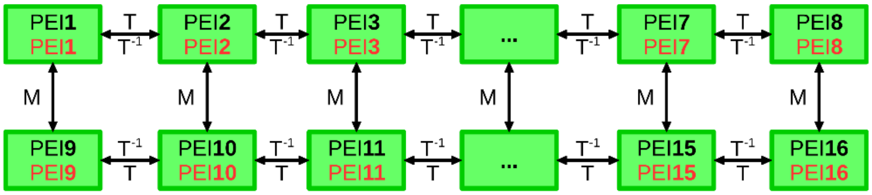

Figure E. (A) The innervation table of the PEI class. (B) The generator diagram. The innervation pattern is very regular and all neurons in the class can be generated by the translation or mirror generators. This class only requires one initial neuron (PEI1)

6. PEN class

The PEN neurons transmit signals from PB to EP (ring P) and NO with a regular innervation pattern (Figure F). No atypical neuron exists in this class. Only one initial neuron is required for generating the entire class of neurons.

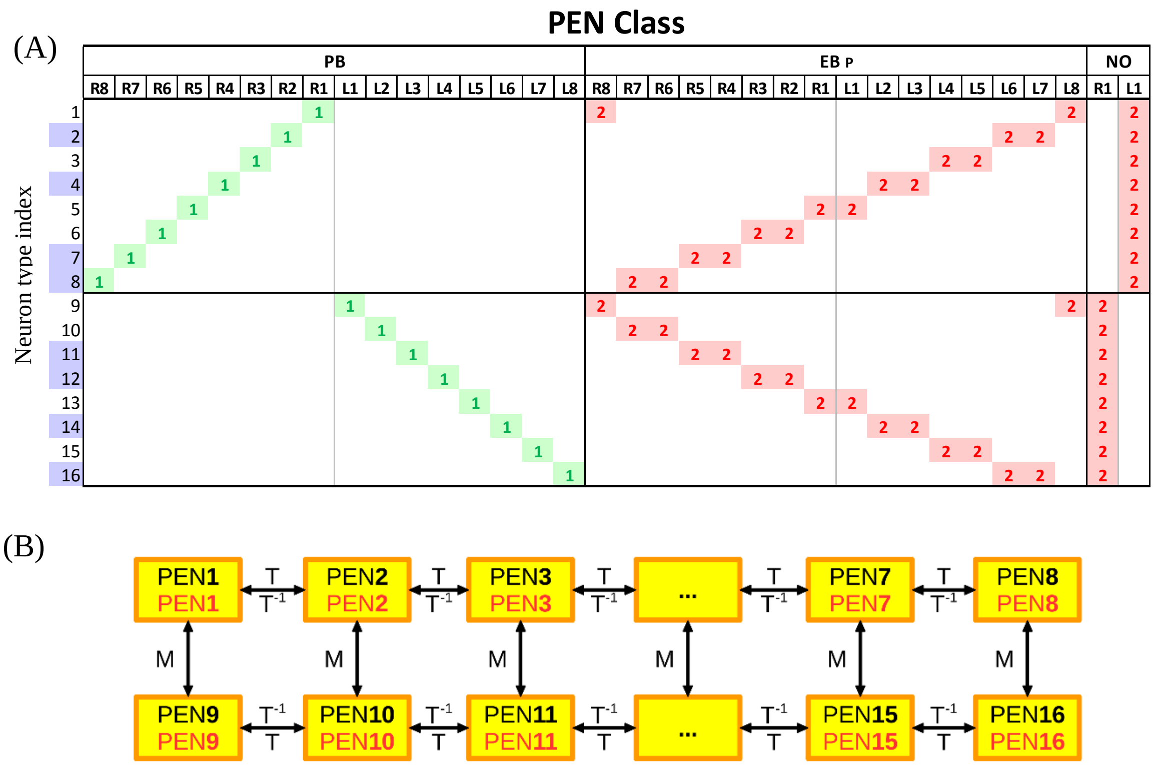

Figure F. (A) The innervation table of the PEN class. (B) The generator diagram. The innervation pattern is very regular and all neurons in the class can be generated by the translation or mirror generators. The PEN class requires only one initial neuron (PEN1).

7. PFN-F_dN_2, PFN-F_eN_3 and PFN-F_fN_4 classes

A large number of neurons in central complex project their axonal terminals to FB and NO with the dendritic domains located in PB. Based on the innervated PB layers and NO subunits, these neurons can be divided into three classes: F_dN_2 (FB d layer, NO R2/L2 subunits), F_eN_3 (FB e layer, NO R3/L3 subunits) and F_fN_4 (FB f layer, NO R4/L4 subunits) (Figure G). Although projecting to different FB layers, these three classes of neurons share the same innervation patterns in PB, FB and NO (Figure G), hence can be described by the generators of the same form. Each of the three class contains two pairs of atypical neurons and can be generated from one initial neuron.

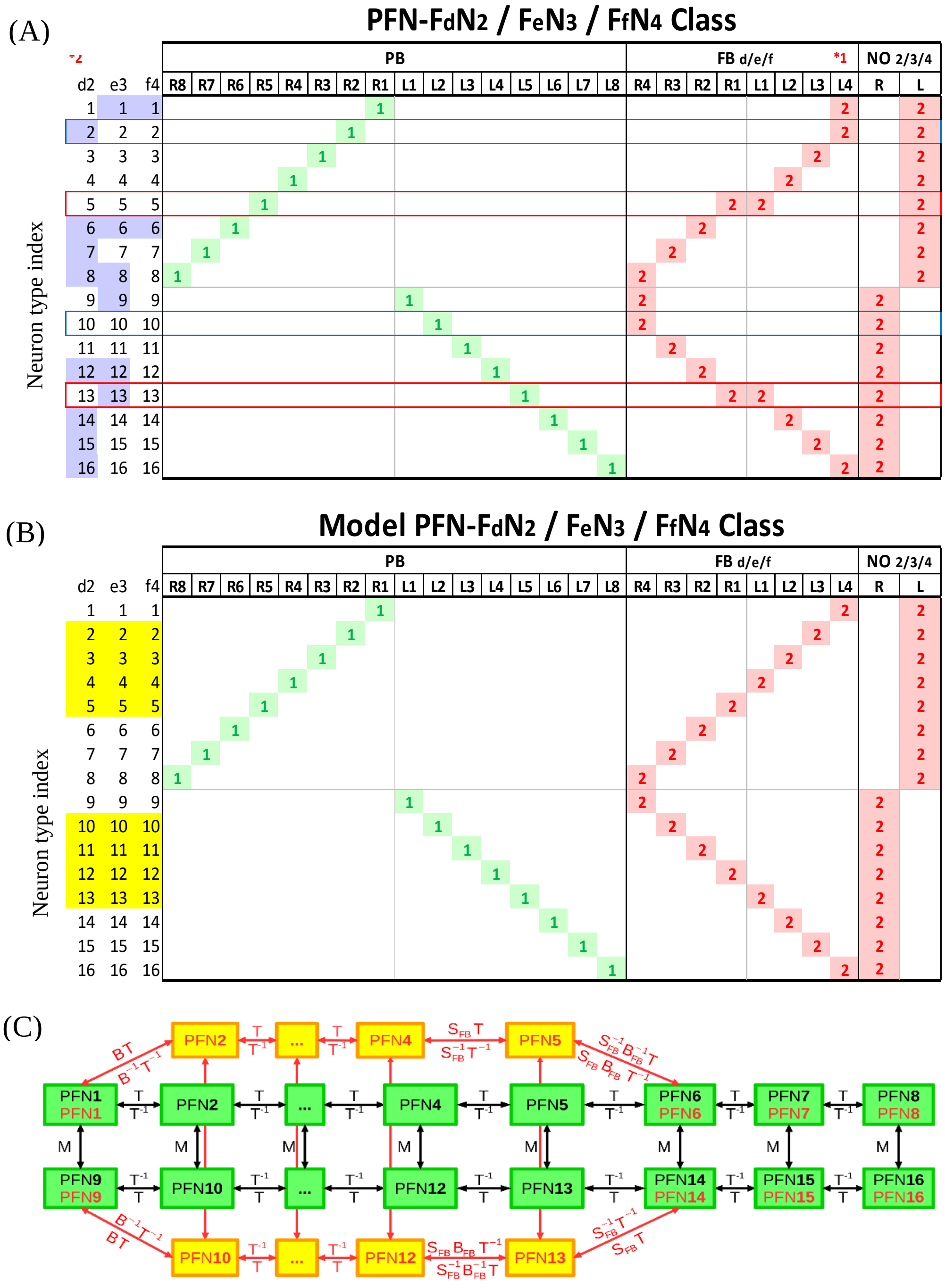

Figure G. The innervation patterns of the three PFN classes in (A) the observed and (B) the model network. (C) The generator diagram. There are two pairs of atypical neurons (types 2 and 10; types 5 and 13) presented in each PFN class. Types 2 and 10 can be described by the backward-translation generator and types 5 and 13 requires a split generator. Both observed and model networks can be generated from only one initial neuron (PFN1).

*1, The subscript d/e/f indicate that each class of the PFN neurons innervate one of the d, e or f layer with the same pattern. Similarly, each class of PFN neurons also innervate one of the regions 2, 3, or 4. *2, d2 indicates the neuron class which innervate d layer in FB and region 2 in NO.

8. PFI-I$_{RB}$ class

The PFI- I$_{RB}$ neurons transmit signals from PB and FB (d and e layers) to the subunits RB in

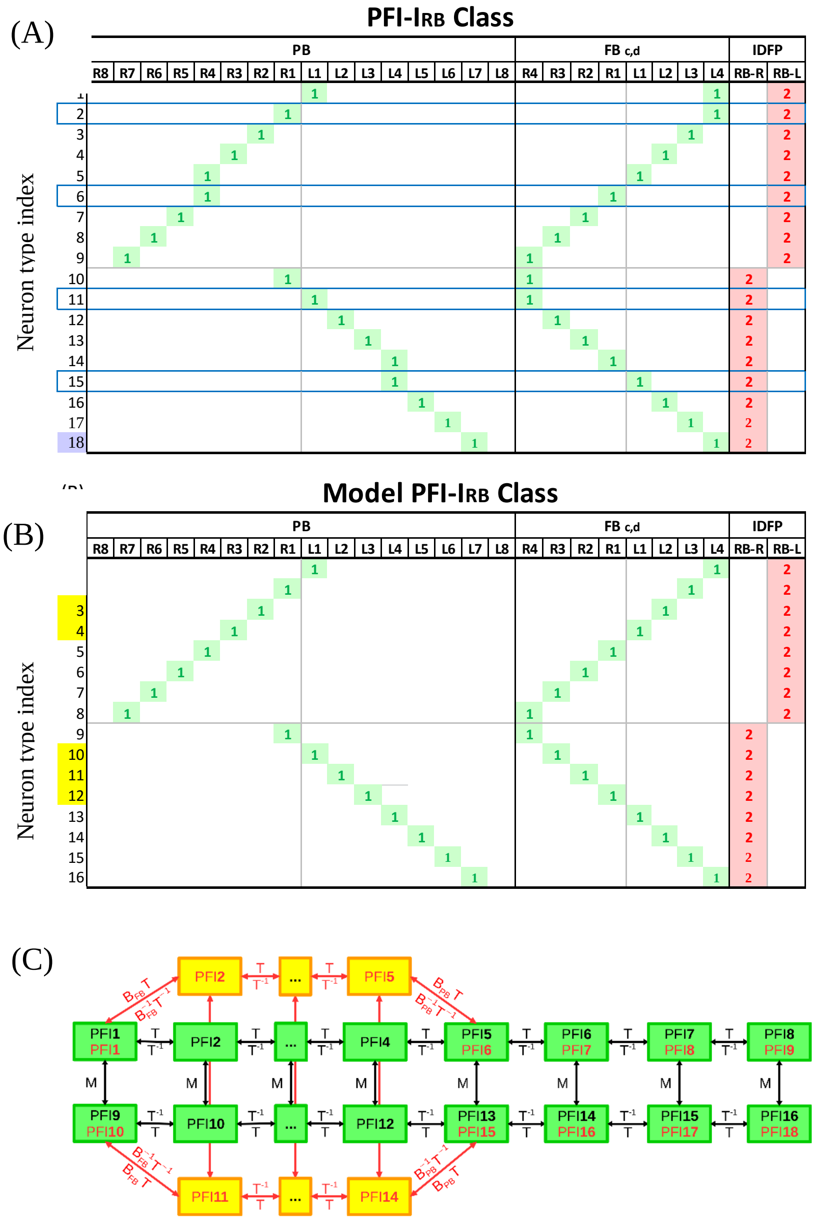

Figure H. The innervation patterns of the PFI-I$_{RB}$ class in (A) the observed and (B) the model network. (C) The generator diagram. There are two pairs of atypical neurons (types 2 and 11; types 6 and 15) which can be described by the backward-translation generator. The class requires only one initial neuron (PFI1) for both observed and model networks.

IDFP (Figure H). The class can be divided into two subclasses: one innervates the c layer and the other innervates the d layer of FB. The two subclasses share the same innervation pattern in the corresponding FB layers. This class possesses two pairs of atypical neurons and can be generated from one initial neuron.

9. PFI-I$_{HBI}$ class

- The PFI-I$_{HBI}$ neurons transmit signals from PB and FB (e layer only) to the subunits HBI in IDFP (Figure I). The class only innervates the e layer of FB and contains two pairs of atypical neurons. Only one initial neuron is required for this class.

(A)

PFI-I$_{HBI}$ Class

Neuron type index	PB																FB e								IDFP	
	R8	R7	R6	R5	R4	R3	R2	R1	L1	L2	L3	L4	L5	L6	L7	L8	R4	R3	R2	R1	L1	L2	L3	L4	HBI-R	HBI-L
1									1														1	1		2
2								1	1														1	1		2
3								1														1	1			2
4							1														1	1				2
5						1														1	1					2
6					1														1	1						2
7				1														1	1							2
8			1														1	1								2
9		1															1	1								2
10								1									1	1							2	
11								1	1								1	1							2	
12									1									1	1						2	
13										1									1	1					2	
14											1									1	1				2	
15												1									1	1			2	
16													1									1	1		2	
17														1									1	1	2	
18															1								1	1	2	

(B)

Model PFI-I$_{HBI}$ Class

Neuron type index	PB																FB e								IDFP	
	R8	R7	R6	R5	R4	R3	R2	R1	L1	L2	L3	L4	L5	L6	L7	L8	R4	R3	R2	R1	L1	L2	L3	L4	HBI-R	HBI-L
1									1														1	1		2
2								1														1	1			2
3							1														1	1				2
4						1														1	1					2
5					1														1	1						2
6				1														1	1							2
7			1														1	1								2
8								1									1	1							2	
9									1									1	1						2	
10										1									1	1					2	
11											1									1	1				2	
12												1									1	1			2	
13													1									1	1		2	
14														1									1	1	2	

(C)

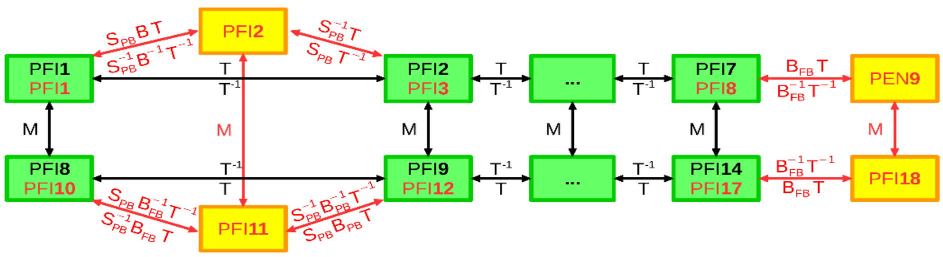

Figure I. The innervation patterns of the PFI-I$_{HBI}$ class in (A) the observed and (B) the model network. (C) The generator diagram. There are two pairs of atypical neurons (types 2 and 11; types 9 and 18) presented in the class. The types 2 and 11 can be generated by applying a backward-translation generator, followed by a split generator while types 9 and 18 only require the application of a backward-translation generator.

10. PFI-I_{HBm} class

The PFI-I_{HBm} neurons transmit signals from PB and FB to the HBm subunits in IDFP. Each neuron in the class innervate two subunits in the c and d layers and one subunit in the e and f layers of FB (Figure J). This class contains two pairs of atypical neurons and requires only one initial neuron. However, the initial neuron of the observed network is different from that of the model network (Figure J).

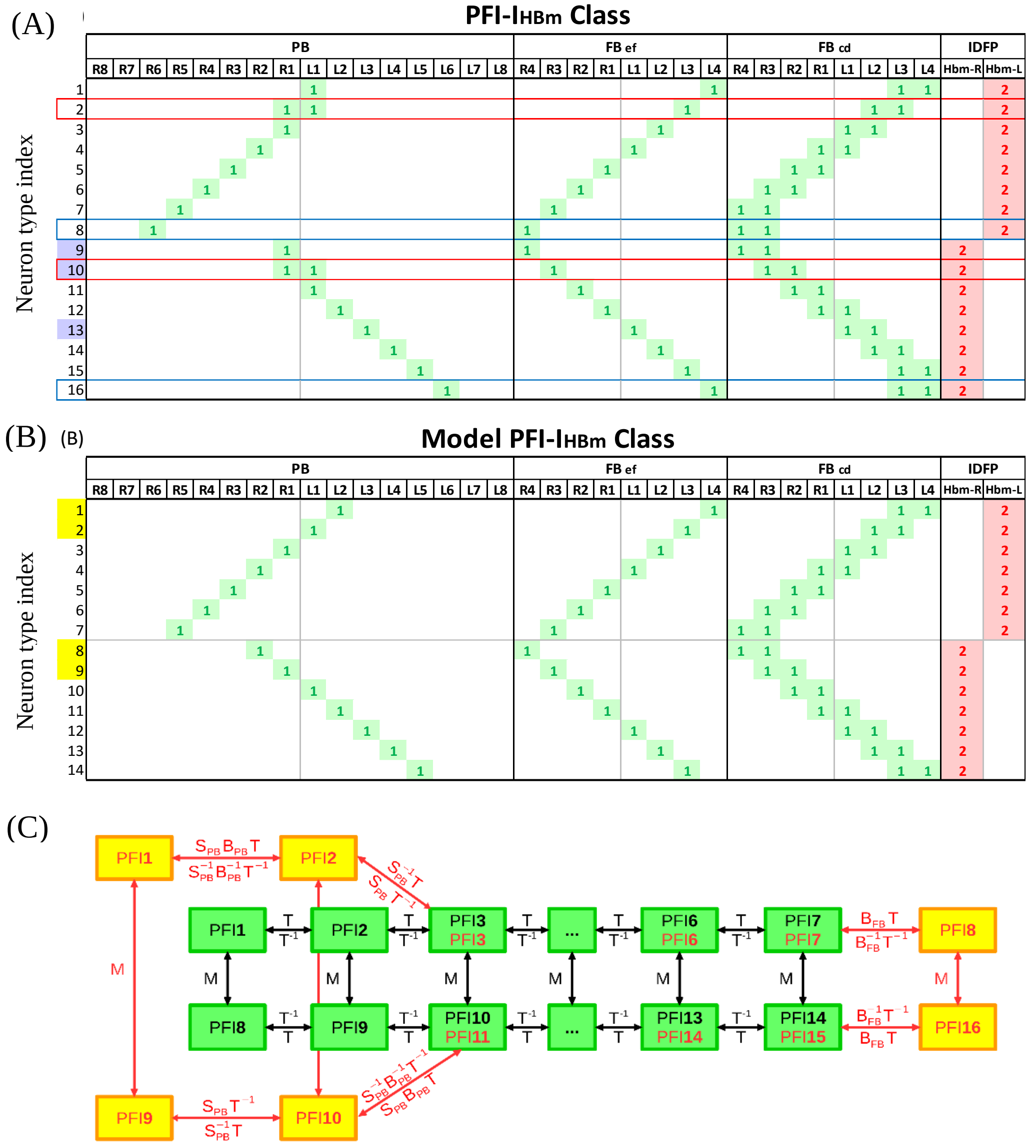

Figure J. The innervation patterns of the PFI-I_{HBm} class in (A) the observed and (B) the model network. (C) The generator diagram. There are two pairs of atypical neurons (types 2 and 10; types 8 and 16) presented in the class. The types 2 and 10 can be generated by applying a backward-translation generator, followed by a split generator while types 8 and 16 only require the application of a backward-translation generator. Note that the initial neurons (PFI 1) are different between the observed and the model networks.

11. PFI-I$_{L+R\text{-}HBm}$

The PFI-I$_{L+R\text{-}HBm}$ class exhibits the innervation patterns that are similar to that of the PFI-I$_{HBm}$ class, except that the former innervates both sides of the IDFP at the same time (Figure K). Only two pairs of the neurons were observed and their innervation patterns in PB and FB do not form a clear translation relationship. We take two ipsilateral neuron types as the initial neurons and generate the other two contralateral neuron types by the mirror generator.

(A)

PFI-I$_{L+R\text{-}HBm}$

Neuron type inde	PB																FB ef								FB cd								IDFP	
	R8	R7	R6	R5	R4	R3	R2	R1	L1	L2	L3	L4	L5	L6	L7	L8	R4	R3	R2	R1	L1	L2	L3	L4	R4	R3	R2	R1	L1	L2	L3	L4	HBm-R	HBm-L
1								1												1							1	1					2	2
2						1											1								1	1							2	2
3									1												1								1	1			2	2
4											1													1							1	1	2	2

(B)

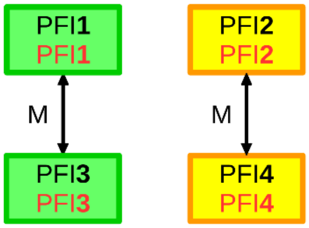

Figure K. (A) Innervation table of the PFI-I$_{L+R\text{-}HBm}$. (B) The generator diagram. The class contains only two pairs of neurons.
