# Supplementary material for: The Topographical Mapping in Drosophila Central Complex Network and Its Signal Routing
Source: Front Neuroinform. 2017 Apr 10;11:26. doi: 10.3389/fninf.2017.00026 (PMC5385387; doi:10.3389/fninf.2017.00026)
Supplement: Supplementary file 7 [file Image1.PDF]

Supplementary Material

Complex Network from Simple Rules – The Topographical Mapping in Drosophila Central Complex Network and its Signal Routing

Po-Yen Chang[#1], Ta-Shun Su[#1], Chi-Tin Shih[*2,3], and Chung-Chuan Lo[*1,4]

[#] These authors contributed equally

*** Correspondence:**

Chung-Chuan Lo: cclo@mx.nthu.edu.tw

Chi-Tin Shih: shih.chi.tin@gmail.com

Supplementary Material Figure

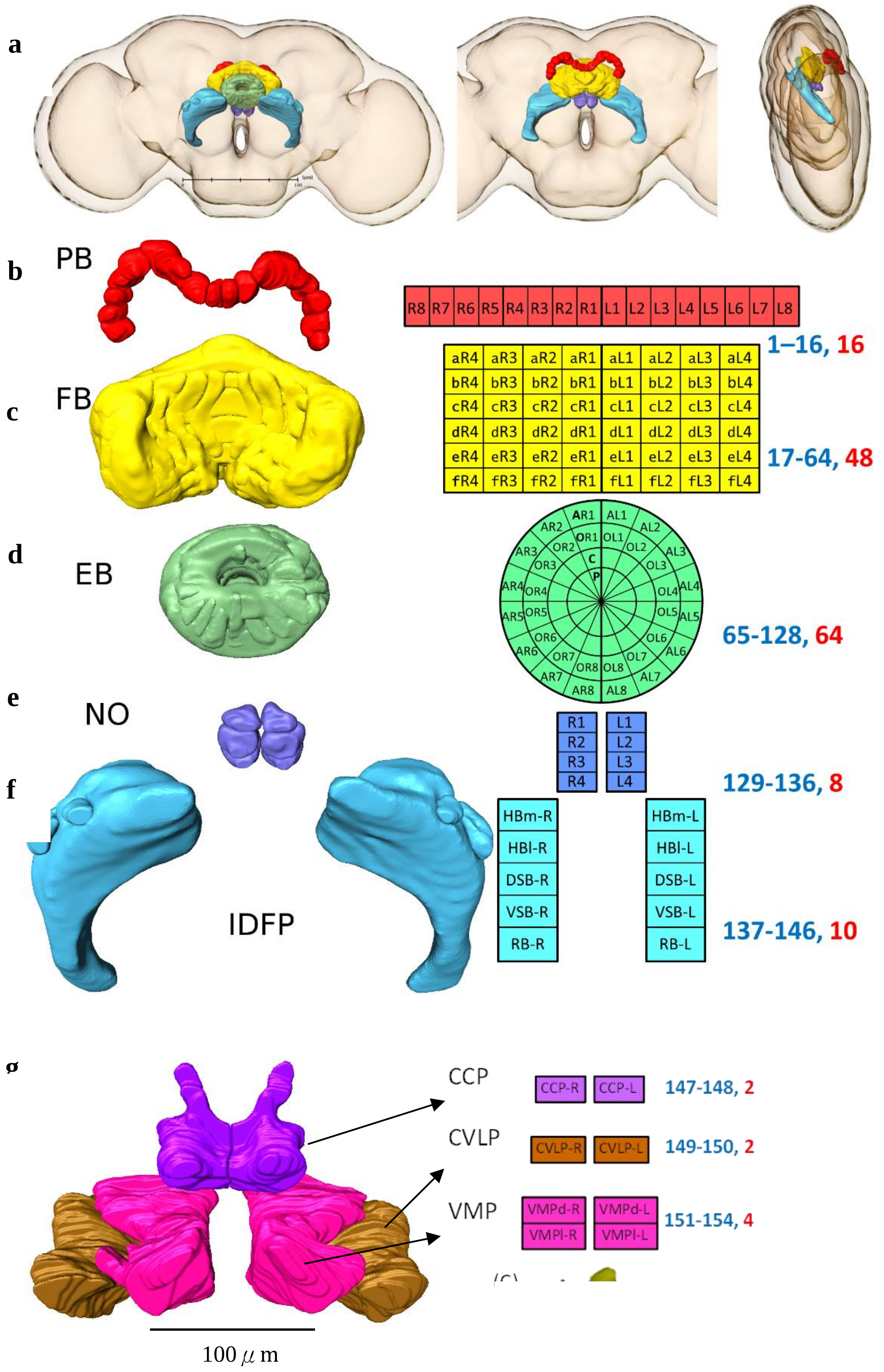

Figure S1. Structure of the central complex and its subunits. a. Anterior (left), posterior (middle), and lateral (right) views of the central complex, which consists of four inter-connected neuropils: protocerebral bridge (PB) (red), fan-shaped body (FB) (yellow), ellipsoid body (EB) (green) and noduli

(NO) (purple). An accessory neuropil, inferior dorsofrontal protocerebrum (IDFP) (light blue), which receives strong innervation from PB-innervating neurons is also shown here. b-e. Detailed view for each neuropil (left) of the central complex and schematics of the subunit division (right). In a recent study (Lin et al 2013), the innervation pattern of each neuron is represented by a 136-dimension vector in which each element in the vector corresponds to a subunit. Red numbers indicate the number of subunits contained in each neuropil, while blue numbers indicate the range of the vector elements allocated for the specified neuropils. f-g. Several associated neuropils outside the central complex also receive innervation from PB-innervating neurons. These neuropils are IDFP (light blue), CCP (purple), CVLP (brown), and VMP (magenta) (left). The four pairs of neuropils are further divided into 18 subunits (right).
