# Supplementary material for: The Topographical Mapping in Drosophila Central Complex Network and Its Signal Routing
Source: Front Neuroinform. 2017 Apr 10;11:26. doi: 10.3389/fninf.2017.00026 (PMC5385387; doi:10.3389/fninf.2017.00026)
Supplement: Supplementary file 8 [file Image2.PDF]

Supplementary Material

Complex Network from Simple Rules – The Topographical Mapping in Drosophila Central Complex Network and its Signal Routing

Po-Yen Chang[#1], Ta-Shun Su[#1], Chi-Tin Shih[*2,3], and Chung-Chuan Lo[*1,4]

[#] **These authors contributed equally**

*** Correspondence:**

Chung-Chuan Lo: cclo@mx.nthu.edu.tw

Chi-Tin Shih: shih.chi.tin@gmail.com

Supplementary Material Figure

PB

R8	R7	R6	R5	R4	R3	R2	R1	L1	L2	L3	L4	L5	L6	L7	L8
1	2	3	4	5	6	7	8	9	10	11	12	13	14	15	16

FB

R4	R3	R2	R1	L1	L2	L3	L4	
17	18	19	20	21	22	23	24	**a**
25	26	27	28	29	30	31	32	**b**
33	34	35	36	37	38	39	40	**c**
41	42	43	44	45	46	47	48	**d**
49	50	51	52	53	54	55	56	**e**
57	58	59	60	61	62	63	64	**f**

EB

R8	R7	R6	R5	R4	R3	R2	R1	L1	L2	L3	L4	L5	L6	L7	L8	
65	66	67	68	69	70	71	72	73	74	75	76	77	78	79	80	**A**
81	82	83	84	85	86	87	88	89	90	91	92	93	94	95	96	**O**
97	98	99	100	101	102	103	104	105	106	107	108	109	110	111	112	**C**
113	114	115	116	117	118	119	120	121	122	123	124	125	126	127	128	**P**

NO

R	L	
129	133	1
130	134	2
131	135	3
132	136	4

IDFP

R	L	
137	142	HBm
138	143	HBl
139	144	DSB
140	145	VSB
141	146	RB

CCP

R	L
147	148

CVLP

R	L
149	150

VMP

R	L	
151	153	d
152	154	l

Figure S2. Subunits in the central complex and associated neuropils. Each subunit is labeled by the number representing the index in the innervation vector.
