# Supplementary material for: The Topographical Mapping in Drosophila Central Complex Network and Its Signal Routing
Source: Front Neuroinform. 2017 Apr 10;11:26. doi: 10.3389/fninf.2017.00026 (PMC5385387; doi:10.3389/fninf.2017.00026)
Supplement: Supplementary file 9 [file Image3.PDF]

Supplementary Material

Complex Network from Simple Rules – The Topographical Mapping in Drosophila Central Complex Network and its Signal Routing

Po-Yen Chang[#1], Ta-Shun Su[#1], Chi-Tin Shih[*2,3], and Chung-Chuan Lo[*1,4]

[#] These authors contributed equally

*** Correspondence:**

Chung-Chuan Lo: cclo@mx.nthu.edu.tw

Chi-Tin Shih: shih.chi.tin@gmail.com

Supplementary Material Figure

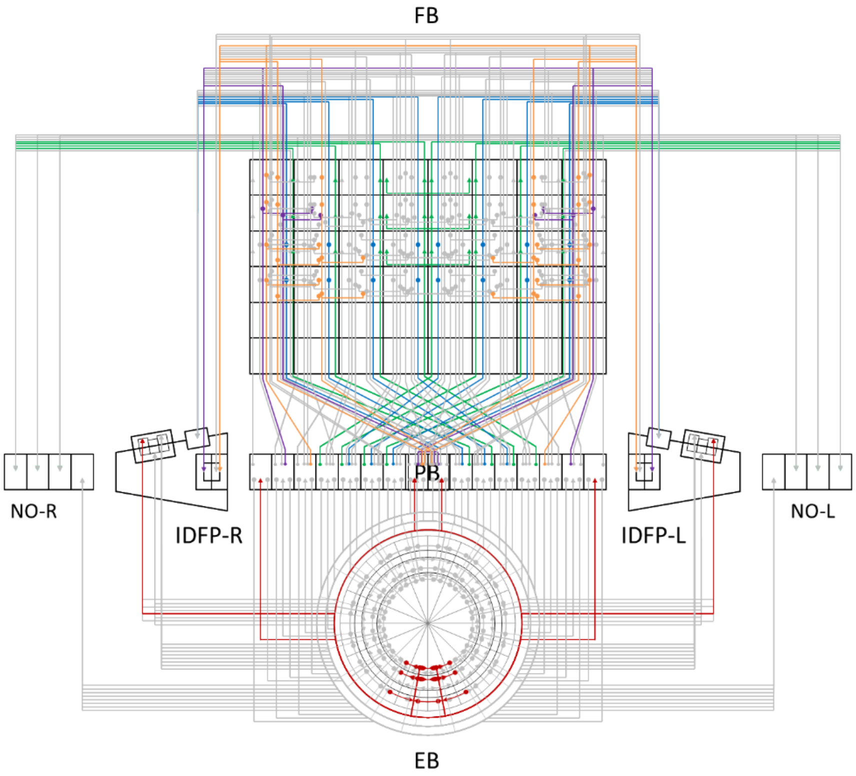

Figure S3. The circuit of the atypical neurons. Out of the 194 neuron types in the observed network, 46 are atypical (color lines) and the rest are typical neurons (grey lines) which are shared by both observed and model networks.
